# GLP-1 and its derived peptides mediate pain relief through direct TRPV1 inhibition without affecting thermoregulation

**DOI:** 10.1038/s12276-024-01342-8

**Published:** 2024-11-01

**Authors:** Eun Jin Go, Sung-Min Hwang, Hyunjung Jo, Md. Mahbubur Rahman, Jaeik Park, Ji Yeon Lee, Youn Yi Jo, Byung-Gil Lee, YunJae Jung, Temugin Berta, Yong Ho Kim, Chul-Kyu Park

**Affiliations:** 1 Gachon Pain Center and Department of Physiology, College of Medicine, Gachon University, Incheon, 21999 Republic of Korea; 2 Department of Anesthesiology and Pain Medicine, Gil Medical Center, Gachon University, Incheon, 21565 Republic of Korea; 3 Lee Gil Ya Cancer and Diabetes Institute Gachon University, Incheon, 21999 Republic of Korea; 4 Pain Research Center, Department of Anesthesiology, University of Cincinnati Medical Center, Cincinnati, OH USA

**Keywords:** Neuropathic pain, Molecularly targeted therapy

## Abstract

Hormonal regulation during food ingestion and its association with pain prompted the investigation of the impact of glucagon-like peptide-1 (GLP-1) on transient receptor potential vanilloid 1 (TRPV1). Both endogenous and synthetic GLP-1, as well as a GLP-1R antagonist, exendin 9–39, reduced heat sensitivity in naïve mice. GLP-1-derived peptides (liraglutide, exendin-4, and exendin 9–39) effectively inhibited capsaicin (CAP)-induced currents and calcium responses in cultured sensory neurons and TRPV1-expressing cell lines. Notably, exendin 9–39 alleviated CAP-induced acute pain, as well as chronic pain induced by complete Freund’s adjuvant (CFA) and spared nerve injury (SNI), in mice without causing hyperthermia associated with other TRPV1 inhibitors. Electrophysiological analyses revealed that exendin 9–39 binds to the extracellular side of TRPV1, functioning as a noncompetitive inhibitor of CAP. Exendin 9–39 did not affect proton-induced TRPV1 activation, suggesting its selective antagonism. Among the exendin 9–39 fragments, exendin 20–29 specifically binds to TRPV1, alleviating pain in both acute and chronic pain models without interfering with GLP-1R function. Our study revealed a novel role for GLP-1 and its derivatives in pain relief, suggesting exendin 20–29 as a promising therapeutic candidate.

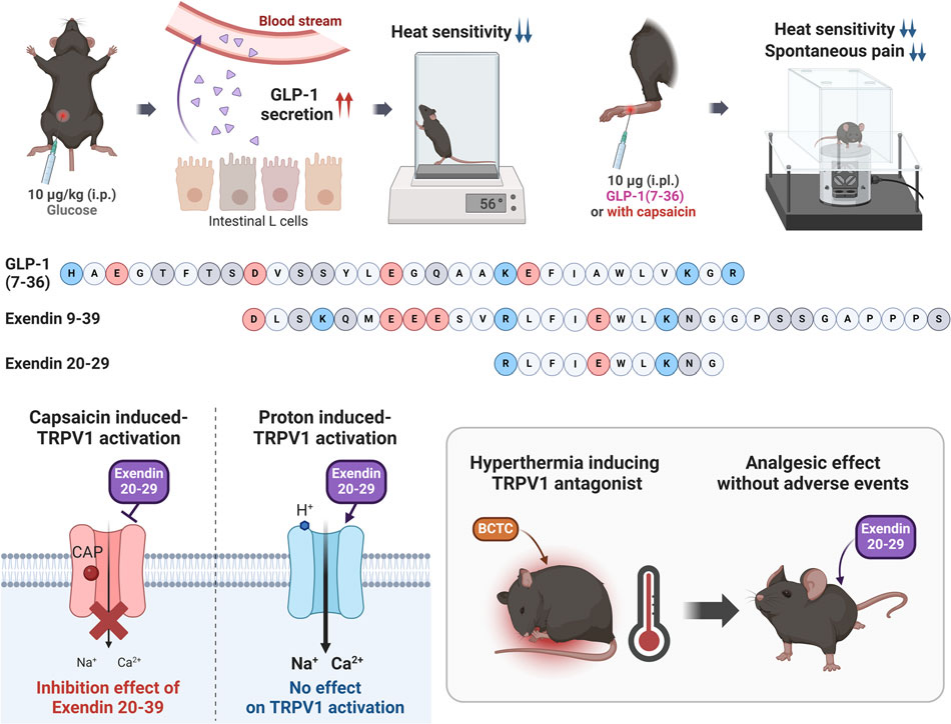

## GLP-1 peptides offer new hope for pain relief

People often consume more sweet and fatty foods when they’re hurting, a pattern observed in both humans and animals. This research investigates how eating can lessen pain, focusing on a hormone named glucagon-like peptide-1 (GLP-1), which is created in the stomach after eating. Researchers carried out tests on mice, using different techniques to see how GLP-1 and its byproducts influence pain perception through their impact on a specific pain receptor located in sensory nerves. The main discovery is that GLP-1 and its byproducts provide a potential new technique for pain relief by directly inhibiting the TRPV1 receptor, which could result in new pain management strategies without the negative effects linked with current treatments. The scientists conclude that targeting the GLP-1 pathway could be a promising approach to developing safer pain medications. This summary was initially drafted using artificial intelligence, then revised and fact-checked by the author.

## Introduction

Individuals experiencing pain often report overeating calorie-dense, high-sugar, and high-fat foods^1^ as a coping mechanism^2^. This phenomenon, termed “ingestion analgesia,” has also been observed in animals^3–6^. Noxious heat-evoked withdrawal behaviors in rats are suppressed during self-initiated chocolate eating and the ingestion of sucrose^7^. Although much research has focused on mechanisms within the central nervous system to explain this pain suppression^8^, we hypothesize that additional mechanisms occurring in the peripheral nervous system are based on hormonal regulation during digestion.

Upon food ingestion and its subsequent entry into the small intestine, intestinal L-cells undergo posttranslational processing of the proglucagon gene, leading to the production of glucagon-like peptide-1 (GLP-1), a potent incretin peptide hormone^9^. GLP-1 plays an important role in glucose homeostasis and is secreted into the hepatic portal system in response to elevated glucose levels from food intake^10^, stimulating insulin synthesis and release from the pancreas^11^. The short half-life of native GLP-1, typically only 1–2 min due to rapid degradation, has facilitated the development of structurally modified GLP-1 analogs with longer half-lives, such as liraglutide, exendin-4, and dulaglutide, which exhibit 97%, 53%, and 90% sequence homology, respectively^12^.

Regarding pain modulation, various studies have reported that GLP-1 analogs exert antinociceptive effects on the central nervous system. This modulation occurs by regulating the spinal dorsal horn microglial pathway through GLP-1R activation, leading to alterations in spinal excitatory synaptic transmission under neuropathic pain conditions^13–18^. However, the effects of GLP-1 on the peripheral nervous system and its relationship with pain modulation remain largely unexplored.

The transient receptor potential vanilloid 1 (TRPV1) channel plays a crucial role in heat pain perception^19^. TRPV1, known for its thermosensitive and polymodal nociceptor properties, is expressed in sensory neurons and is activated by stimuli such as capsaicin (CAP) and protons^20^. In chronic pain states, TRPV1 channels are upregulated in nociceptive neurons, which lowers stimulation thresholds and increases pain perception, as reported in hyperalgesia or allodynia^21^. Inhibiting TRPV1 has proven effective in mitigating pain in diverse neuropathic pain models, thereby garnering interest from numerous pharmaceutical companies^22,23^. However, the challenge with current TRPV1 antagonists lies in their associated adverse effects, such as hyperthermia^24–31^ and hypothermia^32–34^, caused by failures in thermoregulation. This issue has been attributed to the mode of action of these antagonists: those blocking CAP-, proton-, and heat-induced TRPV1 activation result in hyperthermia, whereas those sparing proton-induced activation do not lead to hyperthermia^33,35^. Conversely, the potentiation of proton-induced TRPV1 activation leads to hypothermia^32^.

In light of these challenges, in this study, we assess the impact of GLP-1 and its derivatives on pain behavior and their influence on the peripheral nervous system. Preliminary findings indicate that GLP-1 harbors a key sequence that directly binds to and inhibits the activation of TRPV1 channels in sensory neurons. Importantly, we also demonstrate that GLP-1 and its derivatives directly antagonize TRPV1 channels in a mode-selective manner that can offer pain relief without the adverse thermoregulatory side effects commonly associated with current TRPV1 inhibitors.

## Materials and methods

### Chemicals

CAP and N-(4-tertiarybutylphenyl)-4-(3-cholorphyridin-2-yl) tetrahydropyrazine-1(2H)-carboxamide (BCTC) were purchased from Sigma‒Aldrich (St. Louis, MO), and the stock solutions were prepared with 99.5% ethanol and dimethyl sulfoxide, respectively. AITC and CFA were purchased from Sigma‒Aldrich. GLP-1(7-36), liraglutide, exendin-4, exendin 9–39, exendin 20–29, His-tagged exendin 9–39, and FITC-tagged exendin 9–39 were purchased from Anygen Corp. (Gwangju, South Korea). The stock solutions were prepared according to the supplier’s recommendation for each peptide and were stored at −20 °C. The exendin 20–29 alanine scanning library was purchased from GenScript (Piscataway, NJ).

### Animals

Adult wild-type male C57BL/6 N mice were purchased from Orient Bio (Sungnam, South Korea). The mice were housed at a constant temperature and humidity under a 12-h/12-h light‒dark cycle with free access to standard food and water for at least 1 week prior to the beginning of the experimental procedures. All animal experiments were approved by the Institutional Animal Care and Use Committee of the College of Medicine at Gachon University (approval number: LCDI-2020-0135).

### Cell preparation and transient transfection

The dorsal root ganglia (DRGs) were aseptically removed from 5–8-week-old mice and incubated with collagenase A (0.2 mg/mL; Roche, Basel, Switzerland)/dispase-II (2.4 units/mL; Roche) at 37 °C for 90 min. The cells were mechanically dissociated via gentle pipetting and placed on glass coverslips coated with poly-D-lysine.

DRG cells were then grown in neurobasal medium supplemented with 10% fetal bovine serum (FBS; Gibco, Waltham, MA), 2% B27 supplement (Invitrogen, Carlsbad, CA), and 1% penicillin/streptomycin for 24 h before the experiments. Human TRPV1 (hTRPV1)-expressing CHO K1 cells were cultured in Dulbecco’s modified Eagle’s medium (DMEM) supplemented with 10% FBS, 1% penicillin/streptomycin, and 800 µg/mL geneticin in an incubator with 5% CO_2_ at 37 °C. HEK293 and HEK293T cells were also cultured in the same DMEM but without geneticin. Rat TRPV1 plasmids were labeled with green fluorescent protein for subsequent calcium imaging. cDNA constructs of rat TRPV1 were transiently transfected into cells via Lipofectamine 2000 (Invitrogen). Ca^2+^ imaging was performed one day after transient transfection.

### Patch-clamp recordings

Whole-cell voltage-clamp and cell-attached patch-clamp recordings were conducted at room temperature to measure capsaicin (CAP)- or proton-induced currents in dissociated DRG neurons and hTRPV1-expressing CHO K1 cells, respectively. An EPC10 amplifier (HEKA, Stuttgart, Germany) was utilized for these recordings. Patch pipettes, prepared with a micropipette puller (Narishige, Tokyo, Japan), had resistances of 4–5 MΩ for whole-cell recordings and 6–8 MΩ for cell-attached recordings. The recording chamber, with a volume of 500 µL, was continuously superfused at a rate of 1–2 mL/min. CAP- or low pH-induced currents were recorded at a holding potential of −60 mV. Series resistance was compensated ( > 80%), and leak subtraction was performed. The data acquired were low-pass filtered at 2 kHz, sampled at 10 kHz, and analyzed via PatchMaster and FitMaster (HEKA) software. The internal solution for whole-cell and cell-attached recordings consisted of 126 mM K-gluconate, 10 mM NaCl, 1 mM MgCl_2_, 10 mM EGTA, 10 mM HEPES, 2 mM NaATP, and 0.1 mM Na_2_GTP, adjusted to pH 7.3 with KOH, with an osmolarity of 295–300 mOsm. An external solution of 140 mM NaCl, 5 mM KCl, 1 mM MgCl_2_, 2 mM EGTA, 10 mM HEPES, and 10 mM glucose, adjusted to pH 7.4 with NaOH, osmolarity 300–310 mOsm, was used for both techniques.

Inside-out recordings from hTRPV1-expressing CHO K1 cells were conducted using a pipette resistance of 8–9 MΩ. The internal solution for the inside-out recordings matched that used for the whole-cell and cell-attached methods, whereas the external solution (intracellular side) differed slightly, containing 2 mM CaCl_2_ instead of EGTA. The open probability and average single-channel opening and closing times for the inside-out recordings were analyzed via a 50% threshold criterion, with all events double-checked before analysis.

### Ca^2+^ imaging in dissociated DRG neurons and hTRPV1-CHO K1 cells

At room temperature, Ca^2+^ imaging was conducted in mouse DRG, HEK293T, and hTRPV1-expressing CHO K1 cells. Cells on poly-D-lysine-coated coverslips were loaded with 2 µM Fura-2 AM (Thermo Fisher Scientific, Waltham, MA) at 37 °C for 40 min in DMEM. The cells were then rinsed three times with the medium and incubated for 30 min, after which they were placed on the stage of an inverted microscope (BX51W1; Olympus, Tokyo, Japan) and continuously superfused at a flow speed of 1 mL/min with a bath solution containing 140 mM NaCl, 5 mM KCl, 1 mM CaCl_2_, 2 mM MgCl_2_, 10 mM HEPES, and 10 mM glucose, adjusted to pH 7.4 with NaOH. Using illumination with a 175-W xenon arc lamp, excitation wavelengths (340/380 nm) were selected via a Lambda DG-4 monochromator wavelength changer (Shutter Instrument, Novato, CA). The fluorescence 340/380 ratio was measured via digital video microfluorometry with an intensified camera (OptiMOS; QImaging, Surrey, Canada) coupled to the microscope. The data were analyzed via SlideBook 6 (Intelligent Imaging Innovations, Denver, CO). At the end of the experiment, the cells were identified on the basis of their response to high concentrations of KCl.

### Pull-down assay and immunoblotting

Interactions between exendin 9–39 and TRPV1 were examined via His-mediated pull-down assays using a modified protocol from the Pierce Pull-down PolyHis Protein:Protein Interaction Kit (Thermo Fisher Scientific). His-tagged exendin 9–39 was bound to HisPur Cobalt Resin as the bait protein and incubated with lysates of hTRPV1-CHO K1 or native CHO K1 cells overnight at 4 °C. After washing five times with lysis buffer, the complexes were mixed with lysis buffer containing 290 mM imidazole, and the bound proteins were eluted by boiling in 5× sodium dodecyl sulfate (SDS) loading buffer for 5 min.

The products were then separated via SDS‒polyacrylamide gel electrophoresis, transferred onto nitrocellulose membranes, and blotted with an anti-TRPV1 antibody (#ACC‒030; Alomone Labs, Jerusalem, Israel). The membrane was washed with TBST and incubated for 1 h with the secondary antibody, anti-rabbit Ig-HRP (1:10,000, 9910; Cell Signaling Technology, Danvers, MA). After washing with TBST, the immune complexes were detected via chemiluminescence (Beyotime, Shanghai, China) using a Pierce western blotting kit (Thermo Fisher Scientific). Quantitative densitometric analysis was performed via a UVP BioSpectrum multispectral imaging system (Image Quant LAS 4000; GE Healthcare, Chicago, IL).

### ELISA

The mice were anesthetized using isoflurane, and blood samples were collected at specified time points. Blood was drawn before (0 min) and after the intraperitoneal administration of glucose (2.0 g/kg body weight) at intervals of 2, 6, 10, 30, 60, and 120 min (*n* = 3–4 per time point). Serum was obtained by centrifuging the collected whole blood at 3000 ×g for 15 min at 4 °C. The supernatant was then transferred to a fresh tube, and total GLP-1 levels were measured via an ELISA kit (EZGLP1T-36K, Millipore, Tokyo, Japan) following the manufacturer’s instructions.

### Confocal fluorescence imaging

Approximately 1 × 10^5 ^mL^−1^ of hTRPV1-expressing CHO K1 cells or naïve CHO K1 cells were seeded into confocal dishes and cultured overnight for cell adherence. The cells were washed with phosphate-buffered saline (PBS) and fixed with 2% paraformaldehyde for 10 min at 25 °C. After being washed with PBS, the cells were permeabilized with 0.1% Triton X-100 for 5 min at room temperature and blocked with 3% bovine serum albumin with glycine for 30 min. The cells were incubated with a primary antibody against TRPV1 (1:250; #ACC-030, Alomone Labs) for 1 h at room temperature. After washing with PBS three times, an Alexa Fluor 594-conjugated secondary antibody (1:400; Invitrogen) and Hoechst 33342 (Thermo Fisher Scientific) were added for another hour of incubation at 4 °C. The cells were washed with PBS and incubated with FITC-tagged exendin 9–39 dissolved in cold PBS at a concentration of 10 μM for 30 min at 4 °C. Finally, after washing with cold PBS, images were acquired via a confocal laser-scanning microscope with a 100× oil-immersion objective (LSM 700; Carl Zeiss, Oberkochen, Germany).

### Behavioral tests in mice

Baseline heat sensitivity was assessed to evaluate the systemic effects of glucose or GLP-1(7–36) administration via a hot plate apparatus (Ugo Basile, Italy). The paw withdrawal latency (PWL) to heat was measured after either intraperitoneal or oral gavage administration of glucose (2.0 g/kg body weight) in a volume of 200 μL or the same volume of vehicle (0.9% saline) (*n* = 5 per group). To test the local effects of GLP-1(7–36) and exendin 9–39 on heat sensitivity, PWL was measured via the Hargreaves radiant heat apparatus following intraplantar administration of 10 μg of GLP-1(7–36) or vehicle (0.9% saline) (*n* = 5 per group) in a volume of 10 μL.

To evaluate nociceptive behavior, the time spent licking the paw, the paw withdrawal threshold (PWT) to mechanical stimuli, and the PWL to heat were measured as described previously^36^. Either vehicle, exendin 9–39, exendin 20–29, or BCTC was first injected via the intraplantar route, and 30 min later, an additional injection of exendin 9–39, exendin 20–29, or BCTC in combination with CAP was administered.

Licking time was recorded for 5 min after CAP injection in each group. Mechanical allodynia and thermal hyperalgesia were evaluated in separate experiments in a time-dependent manner. Mechanical allodynia was assessed via von Frey filaments (NC12775-99; North Coast Medical, CA). The 50% PWT was calculated via the updown method. Thermal hyperalgesia was assessed by recording the PWL via the Hargreaves radiant heat apparatus (IITC Life Sciences, Woodland Hills, CA). A cutoff value of 20 s was used to prevent tissue damage. For rectal temperature recording, the rectal temperature was measured with a digital thermometer (Therma‐1; ETI, West Sussex, UK) by inserting a corn oil-soaked flexible bead probe into the rectum after the intraperitoneal administration of 200 μL of vehicle, exendin 9–39 (50 µg/kg), or BCTC 5 mg/kg in wild-type mice (*n* = 6). The acute intraperitoneal glucose tolerance test (IPGTT) was performed in 8-week-old mice. Baseline blood glucose levels were measured, and the wild-type mice were intraperitoneally injected with 200 μL of vehicle, exendin 20–29 (10 μg/kg), or exendin-4 (10 μg/kg) (*n* = 6). After 15 min, the mice were challenged with glucose (2.0 g/kg body weight). Blood glucose levels were measured via an Accu-Chek Performa glucometer (Roche, Mannheim, Germany).

### CFA-induced pain

Under temporal anesthesia with 3% isoflurane, the CFA-induced inflammatory pain model was established via the intraplantar injection of 20 μL of CFA. The mice were then treated with either vehicle, exendin 9–39, or exendin 20–29 in 20 μL, which were administered on the same paw where the CFA was injected. Heat hyperalgesia and mechanical allodynia induced by CFA were assessed via the Hargreaves test and the von Frey test, respectively. Paw thickness was measured in millimeters via a CD-15APX digimatic caliper (Mitutoyo Corporation, Kawasaki, Japan).

### SNI-induced pain

Under continuous anesthesia with isoflurane, mice underwent surgical manipulation to expose the left sciatic nerve by separating the muscle tissue. Upon visualization of the sciatic nerve, the peroneal and tibial nerves were ligated and transected at the lower end of the ligature via silk thread, whereas the sural nerve remained intact. The surgical site was then sutured, and iodine was applied for debridement. After a recovery period of 14 days postsurgery, the mice received either intraplantar or intraperitoneal administration (20 or 200 μL, respectively) of vehicle, exendin 9–39, or exendin 20–29. The heat sensitivity of the neuropathic pain model mice was subsequently assessed via the Hargreaves test.

### Statistical analysis

Statistical analyses were conducted via GraphPad Prism 8 (GraphPad Software, San Diego, CA). All the data are presented as the mean ± standard error of the mean (S.E.M.). Differences between groups were compared via two-tailed unpaired *t* tests for two groups, one-way analysis of variance (ANOVA) followed by Dunnett’s multiple comparison test for multiple groups, or two-way repeated-measures ANOVA followed by the Bonferroni multiple comparison test for multiple groups and time courses. The statistical significance thresholds were *p < 0.05, **p < 0.01, ***p < 0.001, and ****p < 0.0001 (likewise indicated with # and †).

## Results

### Release of GLP-1 by glucose application alleviates heat sensitivity

**Fig. 1:**
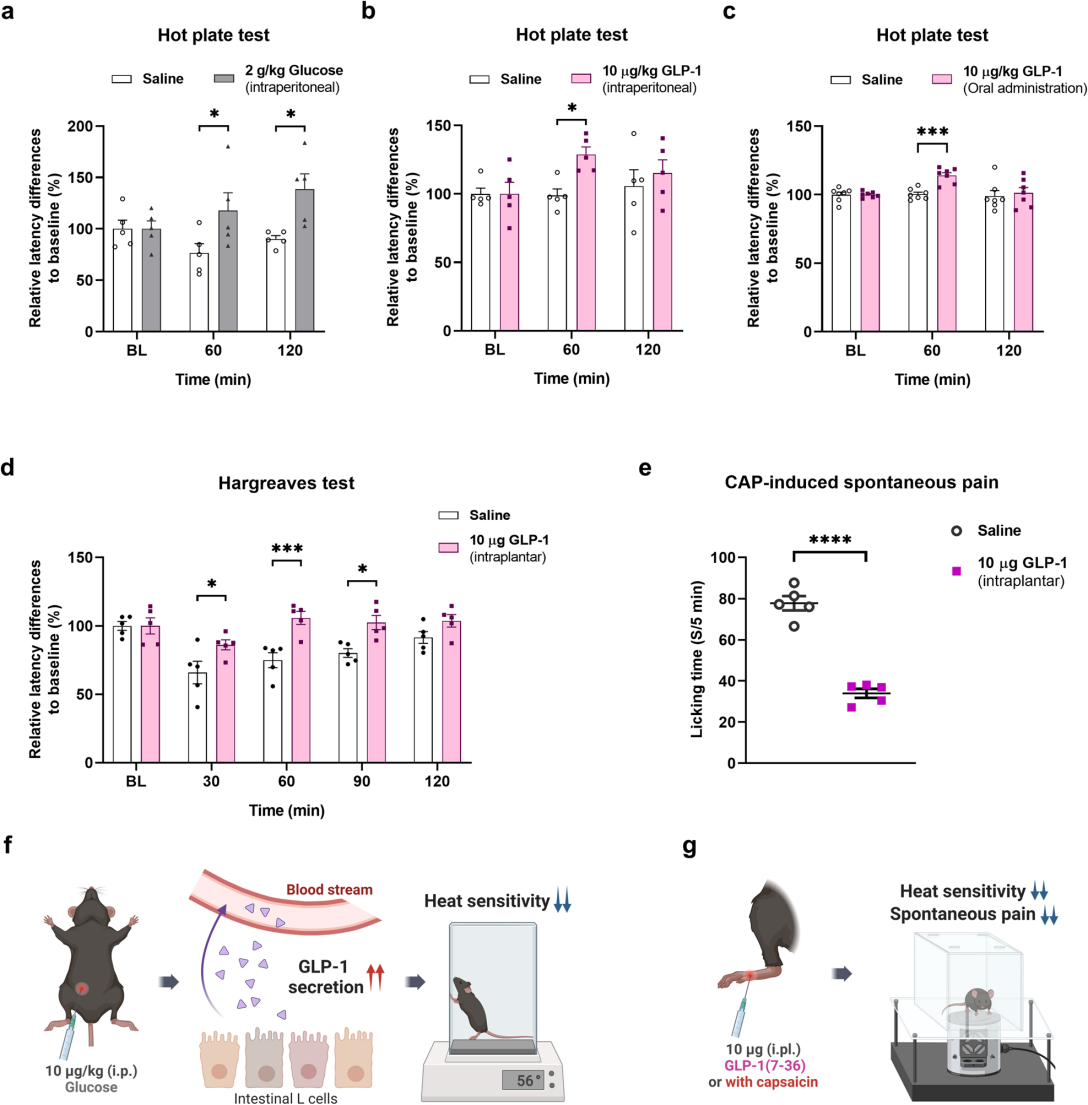
Reduced thermal sensitivity in systemically glucose- or GLP-1 treated- and locally GLP-1 treated-mice and the analgesic effect of GLP-1 on CAP-induced nociceptive behaviors in mice. **a** Effects of intraperitoneal (i.p.) administration of 2 g/kg glucose on heat sensitivity according to the hot plate test (mean ± S.E.M., *n* = 5). Two-way ANOVA followed by the Bonferroni multiple comparison test (*p < 0.05, compared with the saline group). Effects of intraperitoneal (**b**) and oral (**c**) administration of 10 μg/kg GLP-1(7–36) (GLP-1) on heat sensitivity via the hot plate test (mean ± S.E.M., *n* = 5 ~ 7). Two-way ANOVA followed by the Bonferroni multiple comparison test (*p < 0.05, ***p < 0.001, compared with the saline group). **d** Effects of intraplantar administration (i.pl.) of 10 μg of GLP-1(7–36) on heat sensitivity via the Hargreaves test (mean ± S.E.M., *n* = 5). Two-way ANOVA followed by the Bonferroni multiple comparison test (*p < 0.05, ***p < 0.001, compared with the saline group). **e** Effects of intraplantar administration of 10 μg of GLP-1(7-36) on the CAP (1.6 μg)-induced spontaneous licking time (mean ± S.E.M., *n* = 5). Two-tailed unpaired *t* test (****p < 0.0001, compared with the saline group). Schematic illustration of the analgesic effect through systemic GLP-1 secretion (**f**) and local GLP-1 induction (**g**). ANOVA analysis of variance, CAP capsaicin, GLP-1 glucagon-like peptide-1, S.E.M. standard error of the mean.

Given the essential role that GLP-1 plays in regulating glucose levels in the body, we primarily utilized intraperitoneal glucose administration and assessed changes in heat sensitivity in mice via hot plate tests. The role of endogenous GLP-1 in heat sensitivity was demonstrated by the observation that the intraperitoneal glucose-treated group presented a decrease in heat sensitivity compared with the vehicle-treated group (Fig. 1a), as the administration of glucose significantly increased blood glucose levels from 1 to 2 h (Supplementary Fig. 1a). To further explore the relationship between glucose-induced GLP-1 release and heat sensitivity, we examined changes in total GLP-1 levels in the serum following glucose administration (Supplementary Fig. 1b). The data revealed a significant increase in GLP-1 levels, peaking at 30 min postinjection and subsequently decreasing over time. This peak in GLP-1 concentration correlated with the observed changes in heat sensitivity, supporting the hypothesis that the systemic release of GLP-1 contributes to the analgesic effects. To confirm whether the reduction in heat sensitivity after glucose application was due to the systemic release of GLP-1, we intraperitoneally administered 10 µg/kg GLP-1(7–36), one of the two primary biologically active forms of secreted GLP-1. Similar to the results observed with glucose administration, the hot plate test revealed a reduction in heat sensitivity after 1 h (Fig. 1b, f).

Further exploration of GLP-1 administration routes led us to assess the effects of the oral administration of GLP-1(7–36). Remarkably, this method demonstrated a reduction in heat sensitivity that was comparable to, or even exceeded, that observed with intraperitoneal administration (Fig. 1c). This observation underscores the potential of the oral delivery of GLP-1(7–36) as a feasible method for pain prevention. Although our study did not directly evaluate the analgesic effects of oral glucose administration, the analgesic outcomes associated with oral GLP-1(7–36) suggest that increased GLP-1 secretion triggered by oral glucose could similarly influence pain responses. Typically, GLP-1 secretion in response to oral glucose is more pronounced than that in response to other administration routes^37^, suggesting a significant opportunity for future investigations to directly examine the analgesic effects of oral glucose through its impact on GLP-1 levels.

Upon discovering the impact of systemic GLP-1 on heat sensitivity, we evaluated its influence on the peripheral nervous system through local intraplantar injection of 10 µg of GLP-1(7–36). The results of the Hargreaves test demonstrated a reduction in pain sensitivity (Fig. 1d). As changes in heat sensitivity are dependent on TRPV1 activity, we sought to determine whether intraplantar injection of 10 µg of GLP-1(7–36) modulates spontaneous pain behavior induced by the TRPV1 agonist CAP in mice. We found that intraplantar injection of 10 µg of GLP-1(7–36) for 10 min significantly reduced the pain-like (licking) behavior induced by intraplantar injection of 1.6 µg of CAP (Fig. 1e). Therefore, we hypothesized that GLP-1 and its metabolites affect peripheral pain regulation through the TRPV1 channel (Fig. 1g).

### GLP-1 and its analogs modulate CAP-induced TRPV1 activation in mouse DRG neurons

**Fig. 2:**
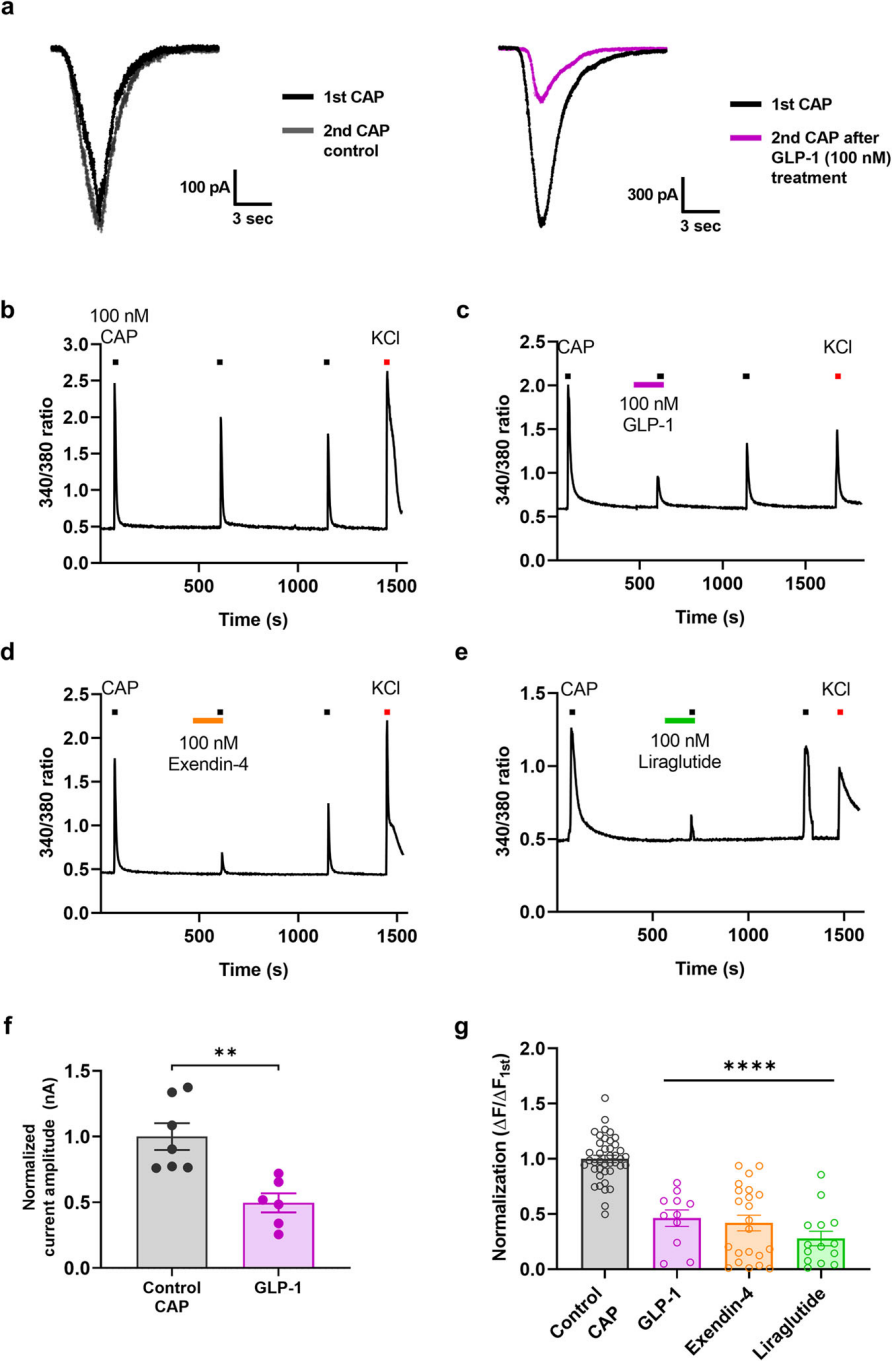
Inhibition of CAP-induced TRPV1 currents and calcium responses in small- to medium-sized mouse DRG neurons following pretreatment with GLP-1 analogs. **a** Representative inward currents induced by 100 nM CAP in the absence (left) or presence of 100 nM GLP-1(7–36) (GLP-1; right). Representative traces of calcium influx elicited by 100 nM control CAP (**b**), GLP-1 (**c**), exendin-4 (**d**), or liraglutide (**e**). The calcium response to high potassium (KCl, 50 mM) was used to identify neurons. **f** Mean normalized currents of sequential CAP-induced currents (mean ± S.E.M.). Two-tailed unpaired *t* test (**p < 0.01, compared with each control CAP). **g** Mean normalized 340/380 ratios of sequential CAP-induced calcium increases (mean ± S.E.M.). One-way ANOVA followed by Dunnett’s multiple comparison test (****p < 0.0001, compared with control CAP). ANOVA analysis of variance, CAP capsaicin, DRG dorsal root ganglia, GLP-1 glucagon-like peptide-1, S.E.M. standard error of the mean, TRPV1 transient receptor potential vanilloid 1.

To determine whether GLP-1 regulates peripheral pain signaling by modulating TRPV1 activity in sensory neurons, whole-cell patch-clamp recordings were performed using small-diameter (<25 µm) neurons dissociated from the mouse DRG. The application of 100 nM CAP elicited inward currents, indicating CAP-responsive DRG neurons, and these responses were abolished by pretreatment with 100 nM GLP-1(7–36) by 52% (Fig. 2a, f). We further tested TRPV1 channel modulation via Ca^2+^ imaging (Fig. 2b–e, g). Pretreatment with each examined GLP-1 analogs, including GLP-1(7–36), liraglutide, and exendin-4, led to significantly reduced Ca^2+^ responses (by 53.8%, 58.1%, and 72.2%, respectively). These results suggest that GLP-1 analogs play an antagonistic role in regulating TRPV1 function.

### The GLP-1 antagonist, exendin 9–39, regulates nociception via TRPV1 modulation

Exendin 9–39, a synthetic peptide, functions as a specific and competitive antagonist of GLP-1^38^. It is derived from exendin-4 through N-terminal truncation and shares 53% sequence homology with native GLP-1^39^.

**Fig. 3:**
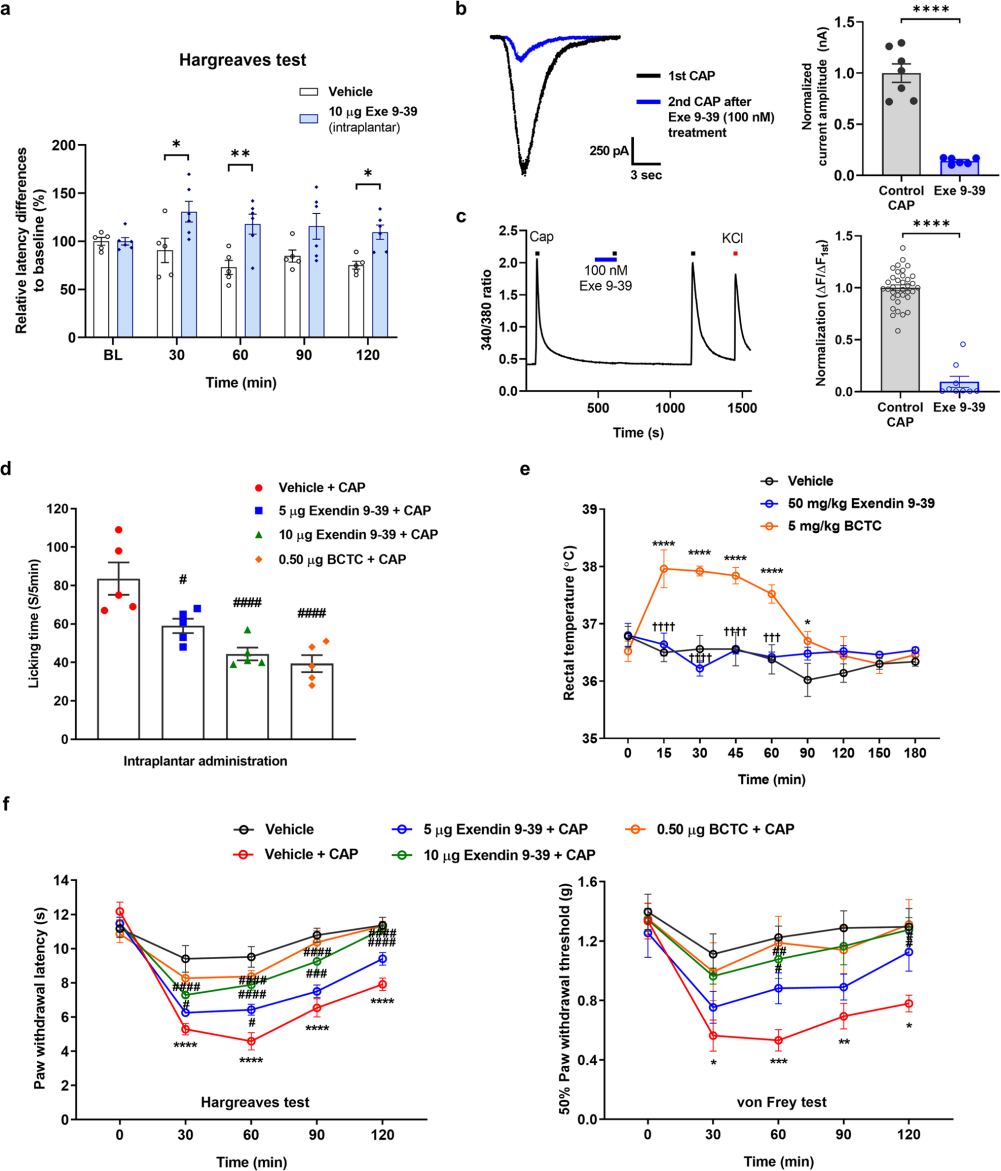
Inhibition of CAP-induced TRPV1 currents and calcium responses in small- to medium-sized mouse DRG neurons following pretreatment with exendin 9–39, an antagonist of GLP-1, and its analgesic effects on CAP-induced nociceptive behaviors in mice. **a** Effects of intraplantar administration of exendin 9–39 (Exe 9–39) (10 μg) on heat sensitivity via the Hargreaves test. Two-way ANOVA followed by the Bonferroni multiple comparison test (*p < 0.05, compared with the saline group). **b** Representative inward currents induced by 100 nM CAP in the presence of 100 nM exendin 9–39 (Exe 9–39; left). Mean normalized currents of sequential CAP-induced currents (mean ± S.E.M.; right). Two-tailed unpaired *t* test (****p < 0.0001, compared with each control CAP). **c** Representative traces of calcium influx elicited by 100 nM control CAP and pretreatment with 100 nM Exe 9–39. The calcium response to high potassium (KCl, 50 mM) was used to identify neurons (left). Mean normalized 340/380 ratios of sequential CAP-induced calcium increases (mean ± S.E.M.; right). Two-tailed unpaired *t* test (****p < 0.0001, compared with each control CAP). **d** Effects of intraplantar administration of exendin 9–39 (5 and 10 μg) and BCTC (0.5 μg) on CAP (1.6 μg)-induced acute licking time (mean ± S.E.M., *n* = 5). One-way ANOVA followed by Dunnett’s multiple comparison test (^#^*p* < 0.05, ^####^*p* < 0.0001, compared with the vehicle + CAP group). **e** Effects of the intraperitoneal administration of exendin 9–39 (50 mg/kg) or BCTC (5 mg/kg) on body temperature (mean ± S.E.M., *n* = 5). Two-way ANOVA followed by the Bonferroni multiple comparison test (*p < 0.05, ****p < 0.0001, vehicle group compared with the BCTC group; ^†††^*p* < 0.001, ^††††^*p* < 0.0001, exendin 9–39 compared with the BCTC group). **f** Effects of intraplantar administration of exendin 9–39 (5 and 10 μg) on CAP-induced acute thermal hyperalgesia (left) and mechanical allodynia (right) in mice (mean ± S.E.M., *n* = 5 each). Two-way ANOVA followed by the Bonferroni multiple comparison test (*p < 0.05, **p < 0.01, ***p < 0.001, ****p < 0.0001, compared with the vehicle group; ^#^*p* < 0.05, ^##^*p* < 0.01, ^###^*p* < 0.001, ^####^*p* < 0.0001, compared with the vehicle + CAP grou*p*). ANOVA analysis of variance, CAP capsaicin, DRG dorsal root ganglia, GLP-1 glucagon-like peptide-1, S.E.M. standard error of the mean, TRPV1 transient receptor potential vanilloid 1.

We initially sought to examine whether exendin 9–39 could reverse the decrease in pain sensitivity caused by local intraplantar injection. However, the results of the Hargreaves test indicated that the administration of 10 µg of exendin 9–39 decreased heat sensitivity upon intraplantar injection (Fig. 3a).

To determine its effects, we pretreated DRG cultures with 100 nM exendin 9–39. Like GLP-1, both CAP-induced TRPV1 inward currents and Ca^2+^ responses were decreased by 85% and 90.6%, respectively (Fig. 3b, c).

To determine the direct effect of exendin 9–39 on the time that mice spent licking their hind paws in the CAP-induced spontaneous pain model, intraplantar administration of exendin 9–39 (doses of 5 and 10 μg) was followed by intraplantar administration of CAP (1.6 μg). Exendin 9–39 dose-dependently reduced the paw licking time, similar to the effect observed with BCTC (0.5 μg), which was used as the control (Fig. 3d). In addition, the paw withdrawal latency (PWL) in the Hargreaves test was significantly lower in the vehicle + CAP group than in the vehicle group from 30 to 120 min (Fig. 3f). Similarly, compared with vehicle injection, intraplantar CAP injection significantly reduced the PWT in the von Frey test from 30 to 120 min compared with that in the vehicle group (Fig. 3f), whereas mechanical allodynia was dose-dependently alleviated in the groups treated with exendin 9–39 (5 or 10 μg) or BCTC (0.5 μg), a widely used TRPV1 inhibitor^40^. These results suggest that the analgesic effects of exendin 9–39 via TRPV1 are similar to those of BCTC.

To determine whether exendin 9–39 treatment causes hyperthermia, which is a typical adverse effect of TRPV1 inhibitors^41^, body temperature was measured following the administration of exendin 9–39. Previous animal studies have shown that the intraperitoneal administration of various TRPV1 antagonists results in hyperthermia^42^; therefore, in this study, exendin 9–39 was injected intraperitoneally. As exendin 9–39 had analgesic effects on the nociceptive behavior of mice at a mass 10 times greater than that of BCTC, the same factor (50 mg/kg exendin 9–39 vs. 5 mg/kg BCTC) was used in this experiment.

The rectal temperature in the group treated with exendin 9–39 was similar to that in the vehicle-treated group. In contrast, the rectal temperature in the BCTC-treated group rapidly increased by 0.7–1.5 °C and returned to baseline values at 12 min postadministration (Fig. 3e).

### Exendin 9–39 alleviates inflammatory and neuropathic chronic pain behaviors

**Fig. 4:**
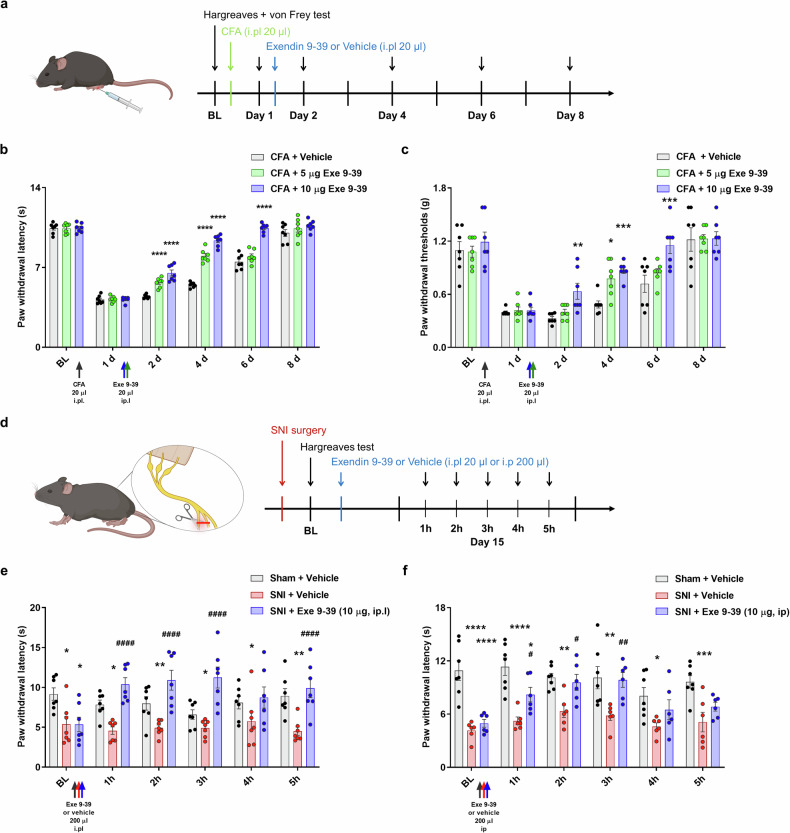
Alleviation of CFA-induced inflammatory and SNI-induced neuropathic pain via exendin 9–39 administration. **a** Schematic illustration and timeline of the CFA-induced inflammatory chronic pain model in mice. **b** Effects of intraplantar injection of exendin 9–39 (Exe 9–39) (5 and 10 μg) on thermal hyperalgesia in a CFA-induced inflammatory chronic pain mouse model via the Hargreaves test (mean ± S.E.M., *n* = 7 each). Two-way ANOVA followed by the Bonferroni multiple comparison test (****p < 0.0001, compared with the CFA group). **c** Effects of intraplantar injection of exendin 9–39 (Exe 9–39) (5 and 10 μg) on mechanical allodynia in a CFA-induced inflammatory chronic pain mouse model via the von Frey test (mean ± S.E.M., *n* = 7 each). Two-way ANOVA followed by the Bonferroni multiple comparison test (*p < 0.05, **p < 0.01, ***p < 0.001, compared with the CFA group). **d** Schematic illustration and timeline of the SNI-induced chronic neuropathic pain model in mice. **e** Effects of intraplantar injection of exendin 9–39 (Exe 9–39) (10 μg) on thermal hyperalgesia in an SNI-induced chronic neuropathic pain mouse model via the Hargreaves test (mean ± S.E.M., *n* = 7 each). Two-way ANOVA followed by the Bo*n*ferroni multiple comparison test (*p < 0.05, **p < 0.01, compared with the Sham + Vehicle group; ^####^*p* < 0.0001, compared with the SNI + Vehicle group). **f** Effects of intraperitoneal injection of exendin 9–39 (Exe 9–39) (10 μg) on thermal hyperalgesia in an SNI-induced chronic neuropathic pain mouse model via the Hargreaves test (mean ± S.E.M., *n* = 7 each). Two-way ANOVA followed by the Bonferroni multiple comparison test (*p < 0.05, **p < 0.01, ***p < 0.001, ****p < 0.0001, compared with the Sham + Vehicle group; ^#^*p* < 0.05, ^##^*p* < 0.01, compared with the SNI + Vehicle grou*p*). ANOVA analysis of variance, CFA complete Freund’s adjuvant, SNI spared nerve injury, GLP-1 glucagon-like peptide-1, S.E.M. standard error of the mean, TRPV1 transient receptor potential vanilloid 1.

The analgesic potential of exendin 9–39 was confirmed in an acute pain mouse model, followed by an assessment of its effectiveness in a chronic pain model. Considering the documented changes in sensitization and upregulation of TRPV1 in the complete Freund’s adjuvant (CFA)-induced inflammatory pain model, CFA was utilized to induce chronic inflammatory pain in mice^43^. The effects of exendin 9–39 on heat hyperalgesia and mechanical allodynia were assessed via the Hargreaves test and the von Frey test, respectively (Fig. 4a). In the Hargreaves test, mice with CFA-induced inflammation displayed delayed recovery from heat hyperalgesia following intraplantar vehicle administration. However, a single intraplantar administration of 5 or 10 μg of exendin 9–39 effectively relieved heat hyperalgesia and accelerated the recovery process (Fig. 4b). Similarly, in the von Frey test, mice injected with the intraplantar vehicle after CFA induction displayed prolonged mechanical allodynia, which was significantly reversed by a single intraplantar administration of 5 or 10 μg of exendin 9–39 (Fig. 4c). Additionally, exendin 9–39 administration mitigated paw swelling resulting from CFA induction (Supplementary Fig. 2).

Furthermore, peripheral nerve injury leads to neuropathic pain, and the spared nerve injury (SNI) model in mice induces persistent heat hyperalgesia^44^. Fourteen days after SNI surgery, compared with those in the sham group, the mice in the SNI group presented heat hyperalgesia after intraplantar vehicle administration (Fig. 4d).

However, SNI-challenged mice administered intraplantar injections of 10 μg of exendin 9–39 presented alleviated heat hyperalgesia starting at 1 h postadministration, peaking at 3 h, compared with those in the vehicle-administered group (Fig. 4e). Similarly, when the same dose of exendin 9–39 was administered intraperitoneally to test its systemic analgesic effects, it alleviated heat hyperalgesia starting at 1 h postadministration, peaking at 3 h, compared with the effects of the intraperitoneal administration of the vehicle (Fig. 4f). These findings suggest that the analgesic effects of exendin 9–39 extend not only to chronic inflammatory pain but also to neuropathic pain.

### GLP-1-derived peptides regulate rat and human TRPV1 activation independently of GLP-1R expression

**Fig. 5:**
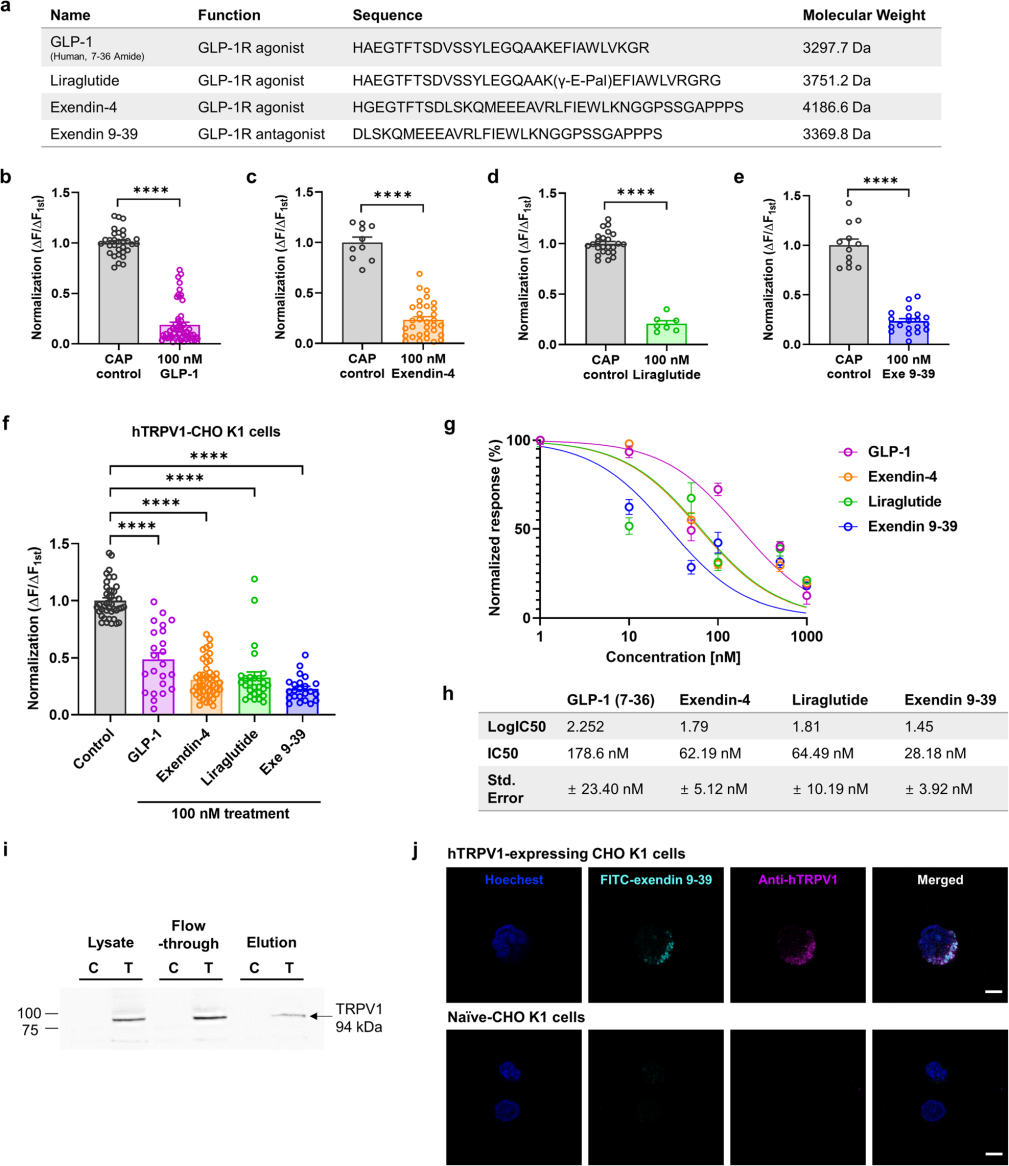
Inhibition of CAP-induced calcium influx via direct interaction with TRPV1 via GLP-1 analogs and exendin 9–39 in HEK293T cells transfected with rat TRPV1 and CHO K1 cells expressing human TRPV1. **a** Sequence alignment, molecular weight, and function of GLP-1 analogs and exendin 9–39. **b**–**e** Mean normalized calcium influx in HEK293T cells transfected with rat TRPV1. Calcium increases were elicited by CAP (100 nM) in the presence of GLP-1 analogs (100 nM each), GLP-1(7–36) (**b**), exendin-4 (**c**), liraglutide (**d**), or exendin 9–39 (Exe 9–39) (**e**) (mean ± S.E.M.). Two-tailed unpaired *t* test (****p < 0.0001, compared with each control CAP). **f** Mean normalized calcium influx in CHO K1 cells expressing human TRPV1. Calcium increases were elicited by CAP (100 nM) in the absence (control CAP) and presence of GLP-1 analogs (100 nM) or exendin 9–39 (100 nM) (mean ± S.E.M.). One-way ANOVA followed by Dunnett’s multiple comparison test (****p < 0.0001, compared with the control, CAP). **g** Curves were fitted via a logistic function to show concentration-dependent inhibition of CAP-induced TRPV1 currents by 100 nM GLP-1, exendin-4, liraglutide, or exendin 9–39. **h** IC_50_ values of GLP-1, exendin-4, liraglutide, and exendin 9–39 (178.6 ± 23.40 nM, 62.19 ± 5.12 nM, 64.49 ± 10.19 nM, and 28.18 ± 3.92 nM, respectively). **i** Pull-down assay using His-tagged exendin 9–39 and cell lysates from CHO K1 cells expressing human TRPV1 (‘T’) and naïve CHO K1 cells (‘C’). Detection of the interaction between exendin 9–39 and TRPV1 via western blotting with an anti-human TRPV1 antibody. **j** Confocal images of FITC-labeled exendin 9–39 in CHO K1 cells expressing human TRPV1 (upper) compared with naïve CHO K1 cells (lower). Overlap of FITC-exendin 9–39 (green), anti-human TRPV1 antibody (red), and Hoechst (blue) in the merged image. Scale bar: 5 µm. ANOVA analysis of variance, CAP capsaicin, CHO K1 Chinese hamster ovary, FITC fluorescein isothiocyanate, GLP-1 glucagon-like peptide-1, S.E.M. standard error of the mean, TRPV1 transient receptor potential vanilloid 1.

To verify the potential involvement of GLP-1R in the observed inhibitory effects on TRPV1 channels, we transiently transfected rat TRPV1 into HEK293T cells. GLP-1R expression was not detected in the HEK293T cell line before transfection (Supplementary Fig. 3). CAP-induced TRPV1 calcium responses were again inhibited by pretreatment with GLP-1 analogs or exendin 9–39 (Fig. 5a–e), indicating that no interaction with GLP-1R was involved. Next, we estimated and compared the half-maximal inhibitory concentration (IC_50_) values from the concentration‒response data using Chinese hamster ovary (CHO K1) cells expressing human TRPV1. The IC_50_ values for the calcium response of GLP-1, liraglutide, exendin-4, and exendin 9–39 were 178.60, 62.19, 64.49, and 28.18 nM, respectively (Fig. 5f–h). To determine the subtype selectivity of exendin 9–39, we tested its effect on TRPA1. Exendin 9–39 did not inhibit or potentiate TRPA1 currents induced by 100 μM allyl isothiocyanate (AITC) (Supplementary Fig. 4a–c), demonstrating that exendin 9–39 is a selective inhibitor of TRPV1. As exendin 9–39 had the lowest IC_50_ value for inhibiting CAP-activated TRPV1 currents, its molecular mechanism was further analyzed.

### Exendin 9–39 directly binds to the TRPV1 channel

To assess the direct interaction between exendin 9–39 and the TRPV1 channel, we conducted a protein binding assay using His-tagged exendin 9–39, which was able to pull down the TRPV1 channel (Fig. 5i) in the CHO K1 cell line stably expressing human TRPV1. In contrast, the naïve CHO K1 cell line showed no TRPV1 channel binding. We tested the binding of exendin 9–39 to TRPV1 by incubating the two cell lines with fluorescein isothiocyanate (FITC)-labeled exendin 9–39. Immunofluorescence imaging revealed that FITC-labeled exendin 9–39 was bound to TRPV1 on the cell surface in CHO K1 cells expressing human TRPV1 but not in naïve CHO K1 cells (Fig. 5j). Although exendin 9–39 was tagged with FITC, it still blocked the CAP-induced inward currents of TRPV1 channels (Supplementary Fig. 5a, b).

### Exendin 9–39 targets the extracellular side of the TRPV1 channel but does not share the CAP binding site

**Fig. 6:**
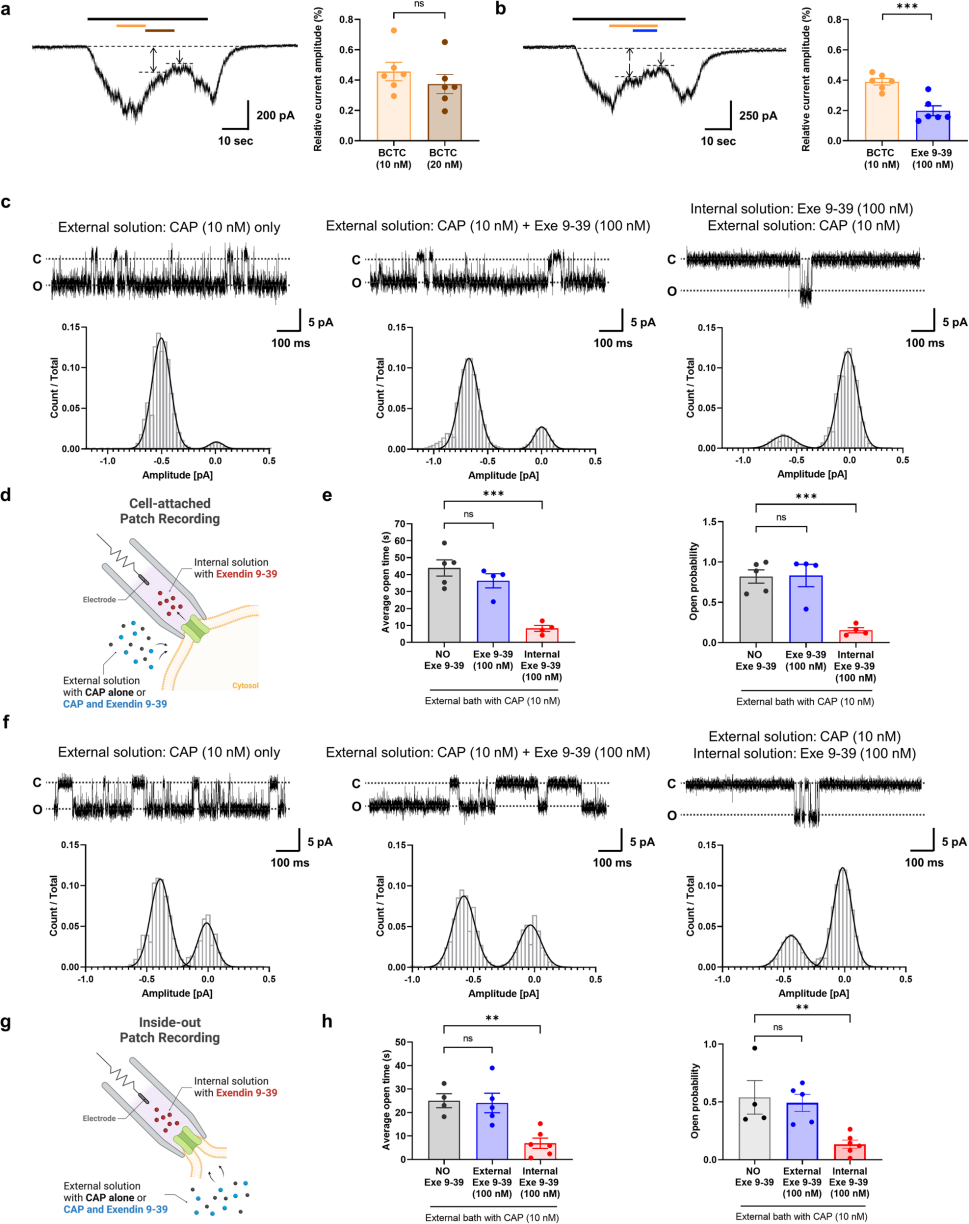
Exendin 9–39 noncompetitively inhibits CAP-induced TRPV1 activation by binding to the extracellular side in CHO K1 cells expressing human TRPV1. **a** Currents evoked by 100 nM CAP were partially blocked by sequential application of 10 and 20 nM BCTC (left). The remaining currents between the arrows were CAP-induced currents inhibited by BCTC. Summary analysis of the inward currents reduced by BCTC alone (mean ± S.E.M.). Two-tailed unpaired *t* test (ns, not significant). **b** Currents evoked by 100 nM CAP were partially blocked by the sequential application of 10 nM BCTC and 100 nM exendin 9–39 (Exe 9–39). The remaining currents between the arrows were CAP-induced currents inhibited by BCTC or exendin 9–39. Summary of inward currents reduced by BCTC alone and BCTC followed by exendin 9–39 (mean ± S.E.M.). Two-tailed unpaired *t* test (***p < 0.001). **c** Representative traces from single-channel recordings of TPRV1 channels in the cell-attached configuration. The cells were held at −60 mV. Exendin 9–39 (100 nM) was delivered via either the external (intracellular) or internal (extracellular) solution in the presence of 10 nM CAP externally. c closed state, o open state. **d** Schematic illustration of cell-attached patch recordings. **e** Average single-channel open time (left) and open probability (right) (mean ± S.E.M.). One-way ANOVA followed by Dunnett’s multiple comparison test (***p < 0.001, compared with no exendin 9–39). **f** Representative traces from single-channel recordings of TPRV1 channels in the inside-out configuration. The cells were held at −60 mV. Exendin 9–39 (100 nM) was delivered via either the external (intracellular) or internal (extracellular) solution in the presence of 10 nM CAP externally. c closed state, o open state. **g** Schematic of inside-out patch recordings. **h** Average single-channel open time (left) and open probability (right) (mean ± S.E.M.). One-way ANOVA followed by Dunnett’s multiple comparison test was used (**p < 0.01, compared with no exendin 9–39). ANOVA analysis of variance, BCTC N-(4-tertiarybutylphenyl)-4-(3-cholorphyridin-2-yl) tetrahydropyrazine-1(2H)-carboxamide, CAP capsaicin, CHO K1 Chinese hamster ovary, S.E.M. standard error of the mean, TRPV1 transient receptor potential vanilloid 1.

Next, we examined the molecular target of exendin 9–39 on the TRPV1 channel via a patch‒clamp competition assay in CHO K1 cells expressing human TRPV1. BCTC, a highly potent TRPV1 antagonist that competitively interacts with CAP to bind to TRPV1 channels^40^, was used for this assay. BCTC (10 nM) was applied in the presence of 10 nM CAP until saturation, and twice the BCTC concentration (20 nM) was sequentially applied. Although 10 nM BCTC blocked the CAP-induced TRPV1 currents, 20 nM BCTC did not significantly potentiate this TRPV1-inhibiting effect (Fig. 6a). In contrast, 100 nM exendin 9–39 application following 10 nM BCTC elicited significant further inhibition of CAP-evoked TRPV1 inward currents (Fig. 6b). These results indicate that exendin 9–39 does not share a binding site with CAP and inhibits TRPV1 activation by interacting noncompetitively with CAP.

To validate the TRPV1 binding site, we performed single-channel recordings from cells expressing human TRPV1 channels. Cell-attached patch recordings revealed that the external bath application of 10 nM CAP and 100 nM exendin 9–39 did not reduce the frequency of single-channel open events. In contrast, when the pipette solution contained 100 nM exendin 9–39, which allowed the interaction of exendin 9–39 with the extracellular surface of TRPV1, single-channel opening events were significantly blocked (Fig. 6c–e). We further conducted inside-out patch-clamp recordings to determine whether bath application of CAP and exendin 9–39 to the intracellular surface of TRPV1 changed single-channel opening events (Fig. 6f–h). The results showed that single-channel openings were blocked by CAP applied to the intracellular side of the channel only when the pipette solution contained 100 nM exendin 9–39, facilitating the exposure of exendin 9–39 to the extracellular surface of TRPV1. In contrast, single-channel opening events were not detected when CAP and exendin 9–39 were administered via the external bath, allowing exposure of CAP and exendin 9–39 to the intracellular surface of TRPV1. Thus, exendin 9–39 binds to the extracellular side of TRPV1 but cannot penetrate the membrane.

### Exendin 9–39 does not affect TRPV1 activation via protons, suggesting its mode selectivity

**Fig. 7:**
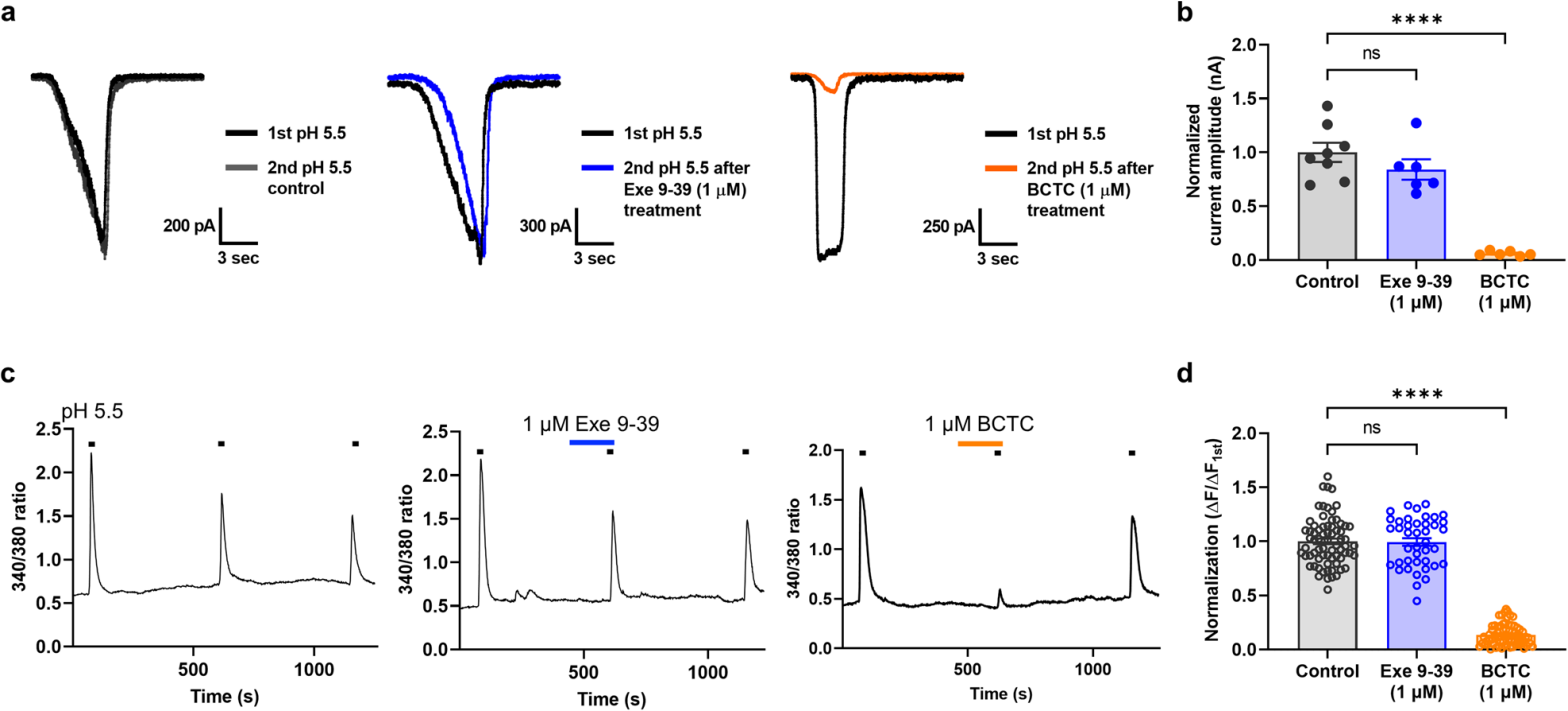
Exendin 9–39 had no effect on proton-induced TRPV1 currents or calcium influx in CHO K1 cells expressing human TRPV1. **a** Representative proton-induced inward currents evoked at pH 5.5 under control conditions (blue) or with 1 µM exendin 9–39 (blue) or 1 µM BCTC (orange) pretreatment. **b** Mean normalized current amplitude of TRPV1 currents (nA) after treatment with 1 µM exendin 9–39 or 1 µM BCTC compared with the control, pH 5.5 (blue) (mean ± S.E.M.). One-way ANOVA followed by Dunnett’s multiple comparison test (ns, not significant; ****p < 0.0001, compared with the control, pH 5.5). **c** Representative proton-induced calcium influx at pH 5.5 under control conditions (blue) or with 1 µM exendin 9–39 (blue) or 1 µM BCTC (orange) pretreatment. **d** Mean normalized 340/380 ratios of sequential proton-induced calcium increases (mean ± S.E.M.). One-way ANOVA followed by Dunnett’s multiple comparison test (ns, not significant; ****p < 0.0001, compared with the control, pH 5.5). ANOVA analysis of variance, BCTC N-(4-tertiarybutylphenyl)-4-(3-cholorphyridin-2-yl) tetrahydropyrazine-1(2H)-carboxamide, CHO K1 Chinese hamster ovary, S.E.M. standard error of the mean, TRPV1 transient receptor potential vanilloid 1.

As the inhibitory effect of exendin 9–39 on CAP-induced activation of TRPV1 was confirmed, its effect on proton-induced activation of TRPV1 was also tested via patch-clamp recordings and calcium imaging. BCTC is also a potent antagonist of TRPV1 activated by protons^45^; therefore, it was used as a control antagonist. Unlike exendin 9–39 during CAP-induced TRPV1 activation, 1 μM exendin 9–39 neither reduced nor potentiated low pH-induced inward currents (Fig. 7a, b) and calcium influx (Fig. 7c, d) in hTRPV1-expressing CHO K1 cells, whereas 1 μM BCTC completely blocked these currents. The exendin 9–39 concentration used in this experiment was approximately 30 times higher than its IC_50_ value for CAP-induced TRPV1 activation, indicating the mode-selective characteristics of exendin 9–39. These findings suggest that exendin 9–39 may be less likely to cause adverse effects associated with thermoregulation, such as hyperthermia or hypothermia, as observed with previous TRPV1 antagonists.

### Small peptides derived from exendin 9–39 inhibit TRPV1 activation and attenuate CAP-induced spontaneous pain in mice

**Fig. 8:**
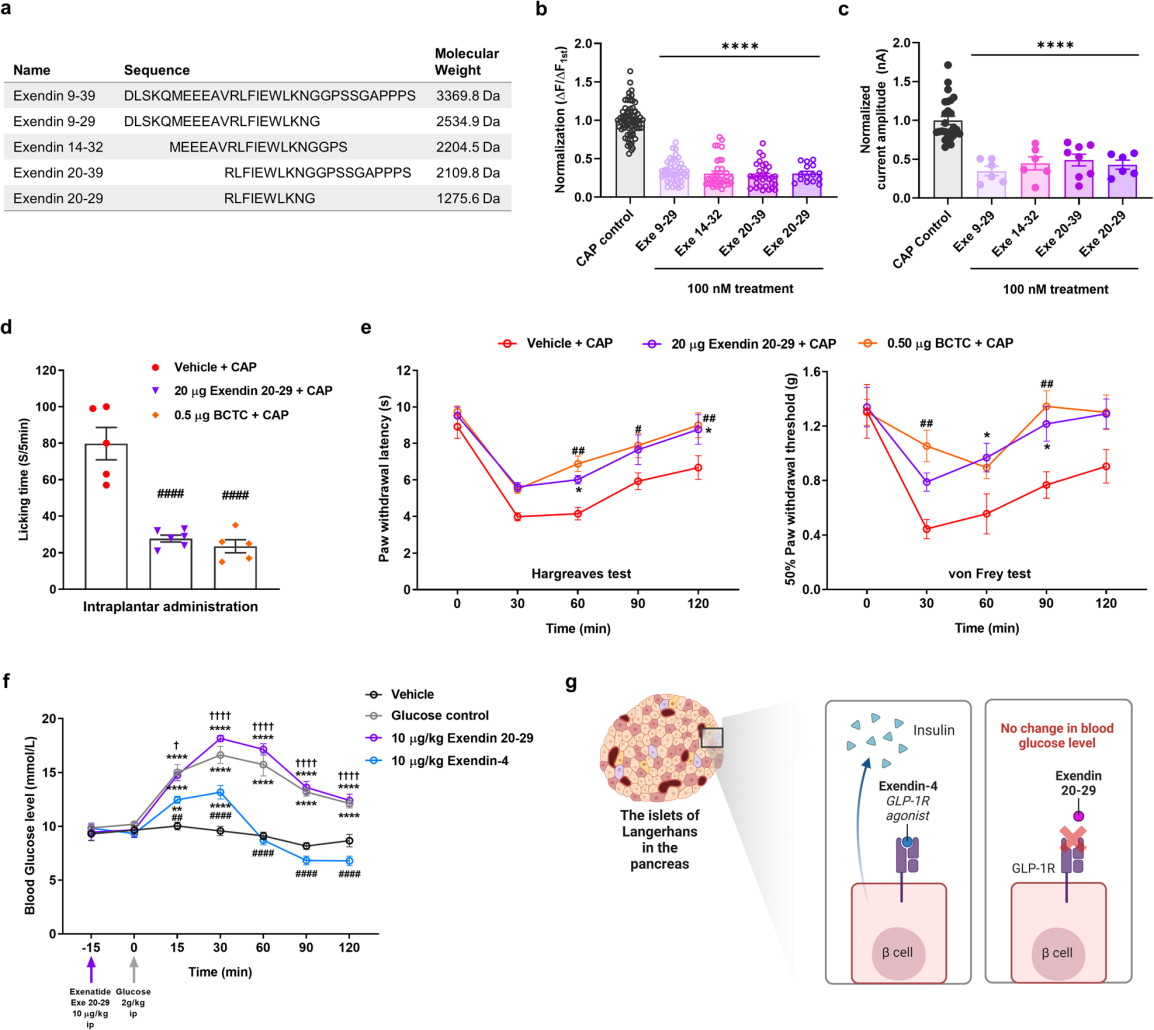
Inhibition of CAP-induced TRPV1 activation by exendin 9–39 fragments and the analgesic effects of exendin 20–29 on CAP-induced nociceptive behaviors in mice. **a** Sequence alignment of three truncated small peptides derived from exendin 9–39, their overlapping sequence exendin 20–29, and the molecular weights of these peptides. **b** Mean normalized calcium influx in CHO K1 cells expressing human TRPV1 with control CAP (100 nM) and each of the four truncated small peptides derived from exendin 9–39 (100 nM each) (mean ± S.E.M.). One-way ANOVA followed by Dunnett’s multiple comparison test (****p < 0.0001, compared with the control, CAP). **c** Mean normalized currents of sequential CAP-induced currents (mean ± S.E.M.). One-way ANOVA followed by Dunnett’s multiple comparison test (****p < 0.0001, compared with the control, CAP). **d** Effects of intraplantar administration of exendin 20–29 (20 μg) and BCTC (0.5 μg) on CAP (1.6 μg)-induced acute licking time (mean ± S.E.M., *n* = 6). One-way ANOVA followed by Dunnett’s multiple comparison test (^##^*p* < 0.01, compared with the vehicle + CAP group). **e** Effects of intraplantar administration of exendin 20–29 (20 μg) on CAP-induced acute thermal hyperalgesia (left) and mechanical allodynia (right) in mice. Two-way ANOVA followed by the Bonferroni multiple comparison test (*p < 0.05, vehicle + CAP group com*p*ared with the exendin 20–29 [20 μg] group; ^#^*p* < 0.05, ^##^*p* < 0.01, vehicle + CAP group com*p*ared with the BCTC [0.5 μg] + CAP) group. **f** Effects of the intraperitoneal administration of 10 μg/kg exendin 20–29 or 10 μg/kg exendin-4 on blood glucose levels following the administration of 2 g/kg glucose (mean ± S.E.M., *n* = 5). Two-way ANOVA followed by the Bonferroni multiple compariso*n* test (**p < 0.01, ****p < 0.0001, compared with the vehicle group; ^##^*p* < 0.01, ^####^*p* < 0.0001, com*p*ared with the glucose control group; ^†^*p* < 0.05, ^††††^*p* < 0.0001, compared with the exendin-4 group). **g** Schematic illustration of the potential mechanism by which exendin 20–29 does not disrupt the regulation of blood glucose levels in the pancreas, where GLP-1 receptors are present. ANOVA analysis of variance, BCTC N-(4-tertiarybutylphenyl)-4-(3-cholorphyridin-2-yl) tetrahydropyrazine-1(2H)-carboxamide, CAP capsaicin, CHO K1 Chinese hamster ovary, GLP-1 glucagon-like peptide-1, S.E.M. standard error of the mean, TRPV1 transient receptor potential vanilloid 1.

To verify the lack of GLP-1R involvement in the observed effects of exendin 9–39, we roughly fragmented exendin 9–39 into three small peptides, i.e., exendin 9–29, 14–32, and 20–39 (Fig. 8a), all of which reduced CAP-induced inward currents (Fig. 8b) and calcium responses (Fig. 8c). Therefore, we assumed that the overlapping peptide sequence exendin 20–29 plays a key role in the inhibition of CAP-induced TRPV1 activation. As hypothesized, exendin 20–29 showed similar inhibitory effects (Fig. 8b, c). Further structural analysis via the program AlphaFold^46^ was conducted to visualize and better comprehend the interactions of the exendin 20–29 sequence with TRPV1 within the broader context of GLP-1 and its derivatives (Supplementary Fig. 6). This analysis highlighted the unique binding potential of exendin 20–29, suggesting its efficacy as a targeted TRPV1 blocker derived from exendin 9–39.

The analgesic effects of exendin 20–29 were then examined in the same manner as those of exendin 9–39. Intraplantar administration of exendin 20–29 (20 μg) also reduced the paw licking time (Fig. 8d). Compared with intraplantar administration of CAP alone (1.6 μg), additional intraplantar injection of exendin 20–29 (20 μg) significantly alleviated pain from 60 to 120 min (Fig. 8e), as manifested by increased PWL in the Hargreaves test. This alleviation was also observed in the group with additional BCTC (0.5 μg) treatment, and no significant differences between the 20 μg exendin 20–29 + CAP and 0.5 μg BCTC + CAP groups were found. Similarly, intraplantar exendin 20–29 (20 μg) injection significantly increased the PWT in the von Frey test from 30 to 90 min compared with CAP (1.6 μg) administration alone (Fig. 8e). However, no significant differences were found between the 20 μg exendin 20–29 + CAP and 0.5 μg BCTC + CAP groups. We also determined whether exendin 20–29 modulates proton-induced TRPV1 activation via patch-clamp recordings and calcium imaging. When 1 μM exendin 20–29 was applied, no significant effect was found, in contrast to the 92% inhibition elicited by 1 μM BCTC (Supplementary Fig. 7a–c). Collectively, these data reveal that exendin 20–29 acts as a key sequence in inducing analgesia in CAP-elicited nociceptive behaviors in mice without causing hyperthermia, and this analgesic effect is mediated by exendin 20–29 directly inhibiting CAP-induced TRPV1 activation but not proton-induced TRPV1 activation.

To determine the potential effects of exendin 20–29 on GLP-1R and glucose regulation, we performed an acute IPGTT in 8-week-old mice. As the GLP-1 analog exendin-4 (10 μg/kg) lowers blood glucose levels^47^, the same amount (10 μg/kg) of exendin 20–29 was administered intraperitoneally. After 15 min of exendin 20–29 or exendin-4 administration, a glucose solution (2 g/kg) was intraperitoneally injected (at 0 min; Fig. 8f). In contrast to exendin-4, which significantly lowered the blood glucose level from 15 to 120 min, exendin 20–29 and the control (without pretreatment) did not change the glucose level during the experiment (Fig. 8f). This result indicates that exendin 20–29 is less likely to interact with GLP-1R (Fig. 8g). Moreover, if the exendin 20–29 mass effective for pain relief (20 μg) was intraperitoneally injected into wild-type mice without glucose treatment, changes in blood glucose levels were not detected. This finding indicates that exendin 20–29 does not activate GLP-1R (Supplementary Fig. 8).

### Exendin 20–29 alleviates inflammatory and neuropathic chronic pain behaviors

Exendin 20–29 was further assessed for its efficacy in alleviating inflammatory and neuropathic chronic pain behaviors. In the CFA-induced chronic inflammatory pain model, mice with CFA-induced inflammation presented prolonged heat hyperalgesia following intraplantar vehicle administration. However, a single intraplantar administration of 20 or 50 μg of exendin 20–29 effectively alleviated heat hyperalgesia, with complete recovery observed by Day 8 (Supplementary Fig. 9a). Similarly, in the von Frey test, mice injected with intraplantar vehicle after CFA induction displayed slow recovery of mechanical allodynia. Nevertheless, a single intraplantar administration of 20 or 50 μg of exendin 20–29 alleviated mechanical allodynia (Supplementary Fig. 9b). Additionally, the administration of exendin 20–29 resulted in a reduction in paw swelling induced by CFA (Supplementary Fig. 9c). Moreover, in the SNI-induced chronic neuropathic pain model, heat hyperalgesia was alleviated in mice challenged with SNI and administered intraperitoneal injections of 50 μg of exendin 20–29, with effects observed as early as 1 hour postadministration and peaking at 2 hours compared with those in the vehicle-administered group (Supplementary Fig. 9d). These findings highlight the therapeutic potential of exendin 20–29 as an analgesic agent for chronic pain management.

## Discussion

Our study identified a novel function for GLP-1-derived peptides in providing pain relief. We specifically found that exendin 20–29 inhibits TRPV1 activity in sensory neurons in a direct and mode-specific manner, reducing pain behaviors without noticeable adverse effects. This discovery is particularly relevant in the context of the ingestion analgesia phenomenon, which is related to the suppression of noxious heat-induced withdrawal behaviors in rats during the consumption of chocolate or sweet liquids, in contrast with salt ingestion^3–7^. A related study revealed that the peripheral administration of D-glucose significantly enhances morphine-mediated antinociception, suggesting a synergistic interaction between the glucose and opioid pathways^48^. Moreover, their findings indicated that D-glucose alone can induce significant antinociceptive responses, independent of opioid medications. These findings suggest a broader role for glucose in pain modulation, which may inform potential therapies targeting both metabolic and pain pathways.

Our primary aim was to dissect the role of the incretin peptide hormone GLP-1 in this context by systemically administering GLP-1 and glucose via intraperitoneal injection. Our findings demonstrated that both natural and synthetic GLP-1 administration altered heat sensitivity and that local administration of GLP-1 notably reduced heat sensitivity and CAP-induced spontaneous pain behavior. These findings suggest that GLP-1 and its metabolites might modulate TRPV1 activity, which plays a pivotal role in heat pain perception^19,49^.

Moreover, our study revealed that GLP-1 analogs, including GLP-1, liraglutide, and exendin-4, along with the GLP-1 antagonist exendin 9–39, derived from exendin-4, which shares 53% sequence homology with native GLP-1^39^, effectively inhibited CAP-induced TRPV1 activation in mouse DRG neurons. Despite lacking the exendin 20–29 sequence RLFIEWLKNG, GLP-1 and liraglutide exhibit analgesic effects, potentially through direct interactions with the TRPV1 receptor. These interactions may be facilitated by sequence homology with peptides such as exenatide and exendin 9–39, which incorporate segments of the exendin sequence. Owing to the rapid degradation of GLP-1 by dipeptidyl peptidase type 4 (DPP-4), it is structurally modified into more stable GLP-1R agonists, such as liraglutide, exenatide, and dulaglutide, which share 97%, 53%, and 90% sequence homology, respectively^12^. This structural modification suggests that similar analgesic effects can be mediated by these GLP-1 analogs, even in the absence of the exendin 20–29 sequence. This concept is further illustrated in Supplementary Fig. 6, which highlights the potential binding sites for each GLP-1 analog in orange, emphasizing their role in pain modulation.

The potential interaction between TRPV1 and GLP-1-derived peptides has been elucidated through structural models. Our results indicate that these peptides modulate TRPV1 function via the extracellular membrane, effectively suppressing activation by CAP without impacting proton-related activation (Supplementary Fig. 10). These findings suggest that the peptides bind to a location that can inhibit the structural changes in TRPV1 caused by capsaicin binding but do not affect the changes caused by salt bridge interactions between proton-sensing residues^50^. Although the exact binding sites remain unclear and have not been directly confirmed by structural analyses, these findings enhance our understanding of how GLP-1-related peptides can selectively modulate TRPV1 without broad systemic effects.

Unlike BCTC, exendin 9–39 also mitigated both thermal and mechanical nociceptive behaviors in mice without inducing hyperthermia, indicating that it is a viable analgesic without the common adverse effects associated with existing pain management strategies, such as opioids and traditional TRPV1 antagonists^32,42,51–53^. Therefore, our study indicated that GLP-1-derived peptides offer effective pain relief without these significant adverse effects, suggesting their potential as safer alternatives for chronic pain management.

Previous studies have shown that GLP-1 analogs can prevent or improve diabetic neuropathy in animal models^54–57^. In addition to their neuroprotective role, GLP-1 analogs have also been studied for their influence on spinal excitatory synaptic transmission under pathophysiological conditions such as neuropathic pain. However, previous studies have not investigated GLP-1 analogs and exendin 9–39 for pain transmission at peripheral sites, especially in small-sized nociceptive DRG neurons, where the TRPV1 channel is crucial in chronic pain-induced mechanical allodynia and thermal hyperalgesia^58–61^.

Recent studies have significantly enhanced our understanding of the role of GLP-1 beyond diabetes management, revealing its substantial involvement in pain modulation. For example, research has shown that the GLP-1 receptor agonist liraglutide can alleviate pain hypersensitivity in chronic migraine models by stimulating interleukin-10, suggesting its potential to modulate central sensitization—a crucial mechanism in chronic pain^62^. Additionally, GLP-1 receptor agonists have demonstrated dual benefits in reducing headache symptoms and promoting weight loss in patients with idiopathic intracranial hypertension, offering therapeutic advantages for conditions involving both metabolic dysregulation and chronic pain^63^. Furthermore, GLP-1 and glucose-dependent insulinotropic polypeptides have been shown to induce substance P release from sensory nerves expressing TRPV1 and TRPA1, broadening the potential pain modulation mechanisms of GLP-1 and suggesting their application in peripheral pain management^64^. Collectively, these findings advance the pharmacological landscape of GLP-1 and its derived peptides, suggesting that they are versatile agents for comprehensive treatment strategies for chronic pain syndromes linked with metabolic disorders.

To identify the mechanism behind the analgesic effects of GLP-1 analogs and exendin 9–39, we utilized a cell line lacking GLP-1R to transfect the rat TRPV1 plasmid. Both GLP-1 analogs and exendin 9–39 were found to reduce CAP-induced inward currents and calcium influx through the TRPV1 channel. Determination of the IC_50_ values for these compounds in CHO K1 cells expressing human TRPV1 revealed that exendin 9–39 had the lowest IC_50_ value for inhibiting CAP-induced TRPV1 activation. These findings suggest that exendin 9–39 directly interacts with TRPV1, independent of GLP-1R, to inhibit its activation. This interaction was further corroborated by pull-down assays and confocal imaging, indicating direct binding between exendin 9–39 and TRPV1 channels. Our electrophysiological analyses revealed that exendin 9–39 likely binds to the extracellular side of TRPV1, offering selective and potent antagonism for pain relief. This finding is particularly significant, as CAP is known to bind to the transmembrane domains of TRPV1 channels^60^, suggesting that exendin 9–39 is a selective and potent antagonist for pain relief without competitively displacing CAP at its binding site.

Although the intraperitoneal administration of exendin 9–39 did not cause adverse hyperthermia effects, a critical aspect of developing TRPV1 antagonists is minimizing interference with TRPV1 activation by protons and hence identifying mode-selective antagonists^32^. Unlike its influence on CAP-induced TRPV1 activation, exendin 9–39 did not block or potentiate proton-induced channel activity, even at a concentration approximately 30 times greater than its IC_50_ value for inhibiting CAP-induced TRPV1 activity, indicating mode-specific inhibition of exendin 9–39.

Critical to our analysis was the examination of the half-maximal effective concentration values for GLP-1 analogs to activate GLP-1R, which are known to be at picomolar levels^65^. To exclude any potential effects of exendin 9–39 on insulin secretion via GLP-1R interaction, we analyzed key protein sequences involved in TRPV1 inhibition. Three distinct fragments of exendin 9–39 were found to reduce CAP-induced inward currents and calcium influx through TRPV1. Their common sequence, exendin 20–29, specifically inhibited CAP-induced TRPV1 activation without affecting proton-induced activation and similarly alleviated CAP-induced nociceptive behaviors. To ensure that exendin 20–29 does not interact with GLP-1R, we utilized an acute IPGTT. Unlike exendin-4, exendin 20–29 did not affect blood glucose levels, supporting its lack of GLP-1R interaction. This finding was further validated by the absence of blood glucose changes in wild-type mice following the administration of exendin 20–29 at effective doses, in contrast with the hypoglycemic effect observed with exendin-4.

Although our results highlight exendin 20–29 as a promising candidate for developing safer peptide analgesics targeting TRPV1, the translation of these findings into clinical practice requires further investigation. This includes a thorough examination of the pharmacokinetics and dynamics in human physiology in comparison with the animal models used in our study. Given the increasing interest in peptide drugs because of their low production costs, high potency, selectivity, and biological stability, our research underscores the potential of finding a peptide analgesic that precisely targets and modulates TRPV1 without causing adverse effects. This represents a promising strategy in the quest for safer and more effective pain management solutions, signaling a potential shift toward innovative analgesic strategies that prioritize both efficacy and patient safety.

## Supplementary information

Supplementary information
