# Supplementary material for: GLP-1 and its derived peptides mediate pain relief through direct TRPV1 inhibition without affecting thermoregulation
Source: Exp Mol Med. 2024 Nov 1;56(11):2449–64. doi: 10.1038/s12276-024-01342-8 (PMC11612315; doi:10.1038/s12276-024-01342-8)
Supplement: Supplementary file 1 — Supplementary information [file 12276_2024_1342_MOESM1_ESM.pdf]

## Supplementary information

# GLP-1 and Its Derived Peptides Mediate Pain Relief Through Direct TRPV1 Inhibition Without Affecting Thermoregulation

## Supplementary Figures

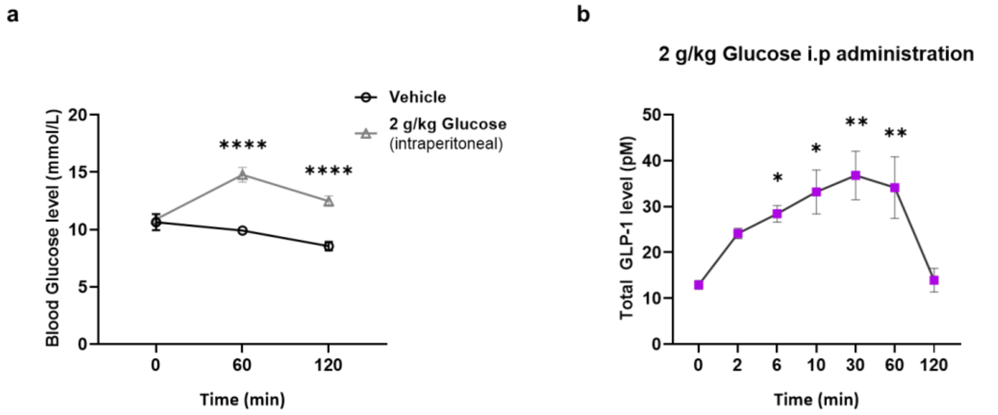


**Supplementary Fig. 1 Increase in blood glucose and total GLP-1 level in serum following intraperitoneal glucose injection in mice. a** Measurement of blood glucose levels following intraperitoneal administration of vehicle or glucose (2 g/kg) in mice. (mean ± S.E.M., *n* = 5). Two-way ANOVA followed by Bonferroni multiple comparison test (****, $p < .0001$, compared with the vehicle group). **b** Serum levels of total GLP-1 measured by ELISA. Blood samples were collected at specified time points following intraperitoneal administration of glucose (2 g/kg) (mean ± S.E.M., *n* = 3 ~ 4). One-way ANOVA followed by Dunnett's multiple comparison test (*, $p < 0.05$, **, $p < 0.01$, compared with 0 min baseline). S.E.M: standard error of mean.

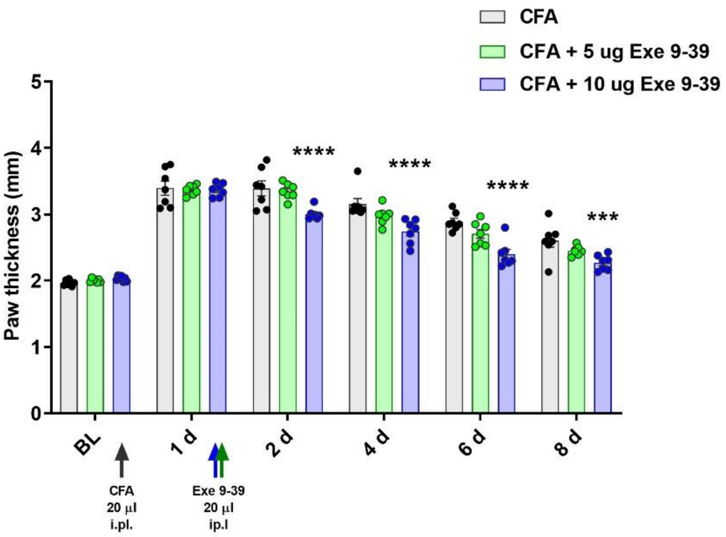


**Supplementary Fig. 2 Reduced paw swelling and thickness via exendin 9–39 administration in CFA-induced inflammatory pain mouse model.** Measurement of the hind paw thickness (mm) on days 1, 2, 4, 6, and 8 after intraplantar administration of exendin 9–39 (Exe 9–39) (5 and 10 μg) (mean ± S.E.M., *n* = 7). Two-way ANOVA followed by Bonferroni multiple comparison test (****p* <.001, *****p* <.0001, compared with the CFA group). ANOVA: analysis of variance, CFA: complete Freund's adjuvant, S.E.M: standard error of mean.

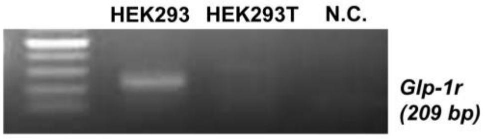


**Supplementary Fig. 3 RT-PCR results showed no expression of GLP-1R in HEK293T cells.** Identification of GLP-1R mRNA expression in HEK293 cells, HEK293T cells, and negative control (N.C.; primer and ultrapure water) using RT-PCR. HEK293 and HEK293T cells were sonicated in TriZol reagent (Invitrogen, Carlsbad, CA), and each RNA was isolated using TriZol and chloroform extraction. Each cDNA was prepared from total RNA via reverse transcription (Superscript Ⅲ, Invitrogen). cDNA was used in separate PCRs. The following primer pairs were used for human GLP-1R: forward (5′-CTCTGGCTGCAGAAGGACAA-3′), reverse (5′-TAGTTCCTGGTGCAGTGCAG-3′).

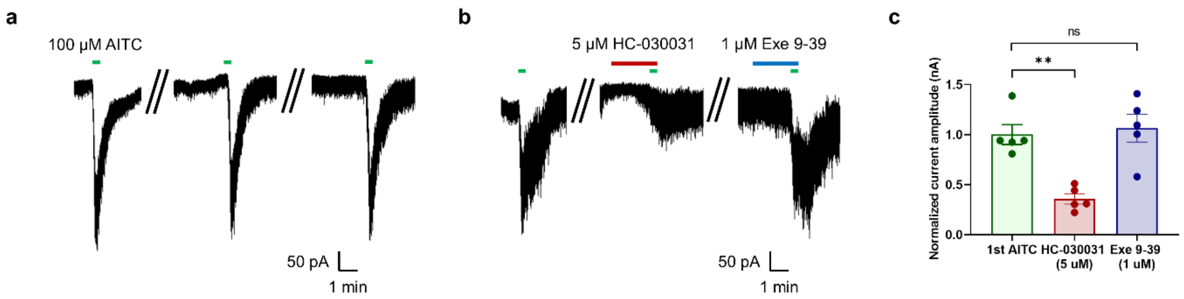


**Supplementary Fig. 4 Exendin 9–39 pretreatment showed no effect on TRPA1 channel activity.** **a–b** Representative inward currents induced by 100 μM AITC without (**a**) or with (**b**) pretreatment with 5 μM HC-030031 or 1 μM exendin 9–39. **c** Mean normalized currents of sequential AITC-induced currents (mean ± S.E.M.). One-way ANOVA followed by Dunnett's multiple comparison test (**$p < 0.01$, compared with the first AITC treatment). ANOVA: analysis of variance, AITC: allyl isothiocyanate, S.E.M: standard error of mean.

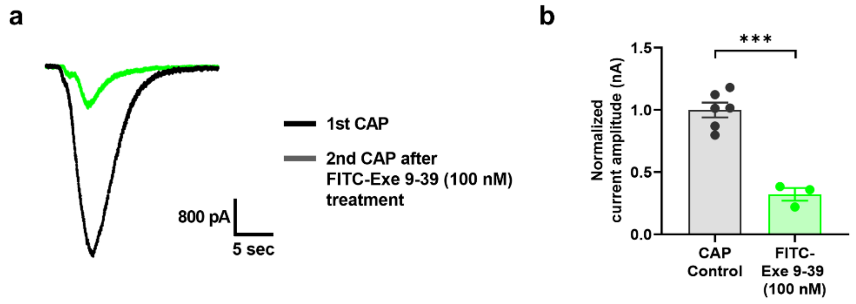


**Supplementary Fig. 5 Functional verification of the inhibitory effect of 100 nM exendin 9–39 on CAP-induced TRPV1 currents in CHO K1 cells expressing human TRPV1. a** Representative CAP-induced inward currents evoked by 100 nM CAP (red) and 100 nM FITC-tagged exendin 9–39 (green) pretreatment. **b** Mean normalized current amplitude of TRPV1 currents following pretreatment with 100 nM FITC-tagged exendin 9–39 compared with control CAP administration (mean ± S.E.M.). Two-tailed unpaired *t*-test (****p* <.001, compared with control CAP). ANOVA: analysis of variance, CAP: capsaicin, CHO K1: Chinese hamster ovary, FITC: fluorescein isothiocyanate, S.E.M: standard error of mean, TRPV1: transient receptor potential vanilloid 1.

**a**

```
GLP-1           HAEGTFTSDVSSYLEGQAAKEFIAWLVKGR---------
Liraglutide     HAEGTFTSDVSSYLEGQAAKEFIAWLVKGRG--------
Exendin-4       HGEGTFTSDLSKQMEEEAVRLFIEWLKNGGPSSGAPPPS
Exendin 9-39    --------DLSKQMEEEAVRLFIEWLKNGGPSSGAPPPS
Exendin 20-29   -------------------RLFIEWLKNG----------
                                  * k̃: K(γ-E-Pal)
```

**b**

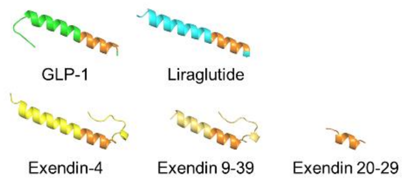


**Supplementary Fig. 6 Structural analysis of GLP-1 and its derived peptides. a** Sequence alignment of GLP-1, liraglutide, and exendin-4 and its analogs exendin 9–39 and exendin 20–29. The region corresponding to exendin 20–29 is highlighted in orange. Liraglutide's modification is denoted where K* indicates a lysine residue linked to a γ-Glu-EO-Palmoate group. **b** Structural models of the peptides were constructed using AlphaFold. These models show the helical configurations maintained across the sequences with the Exendin 20–29 region distinctly emphasized in orange.

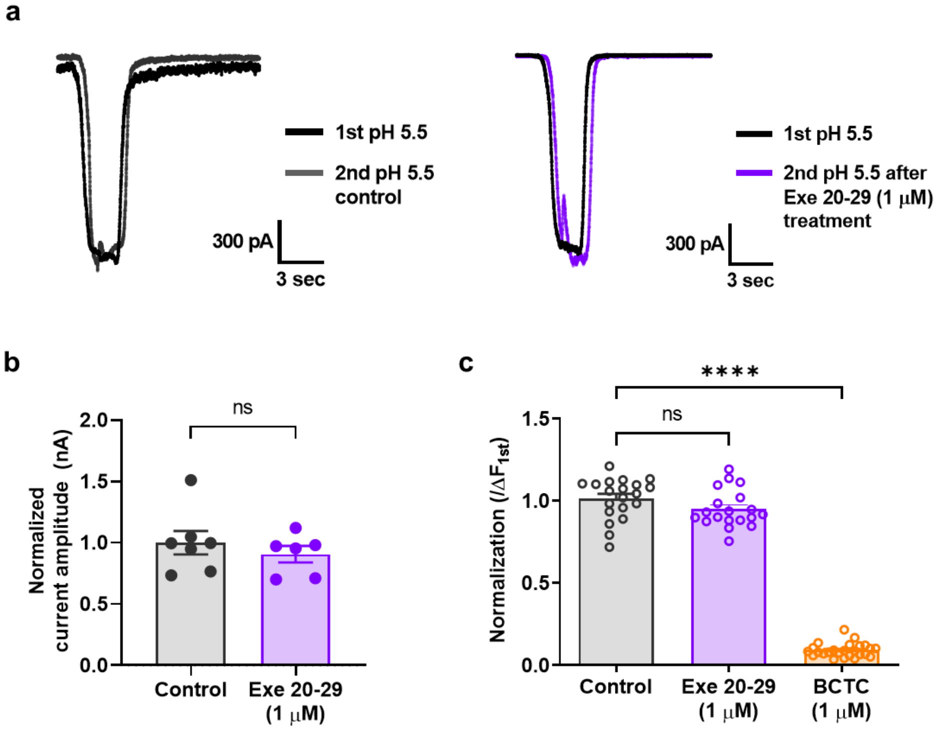


**Supplementary Fig. 7 Exendin 20–29 (1 μM) showed no effect on proton-induced TRPV1 currents and calcium influx in CHO K1 cells expressing human TRPV1. a** Representative proton-induced inward current evoked by control pH 5.5 without (black) or with 1 μM exendin 20–29 pretreatment (purple). **b** Mean normalized current amplitude of TRPV1 currents elicited by pH 5.5 following pretreatment with 1 μM exendin 20–29 compared with control (mean ± S.E.M.). Two-tailed unpaired *t*-test (ns, not significant, compared with control pH 5.5). **c** Mean normalized 340/380 ratio of sequential proton-induced calcium increases following pretreatment with 1 μM exendin 20–29 or 1 μM BCTC (orange), compared with control pH 5.5 (mean ± S.E.M.). One-way ANOVA followed by Dunnett's multiple comparison test. ns, not significant. ****$p$ <.0001, compared with control pH 5.5. ANOVA: analysis of variance, BCTC: N-(4-tertiarybutylphenyl)-4-(3-cholorphyridin-2-yl) tetrahydropyrazine-1(2H)-carboxamide, CHO K1: Chinese hamster ovary, S.E.M: standard error of mean, TRPV1: transient receptor potential vanilloid 1.

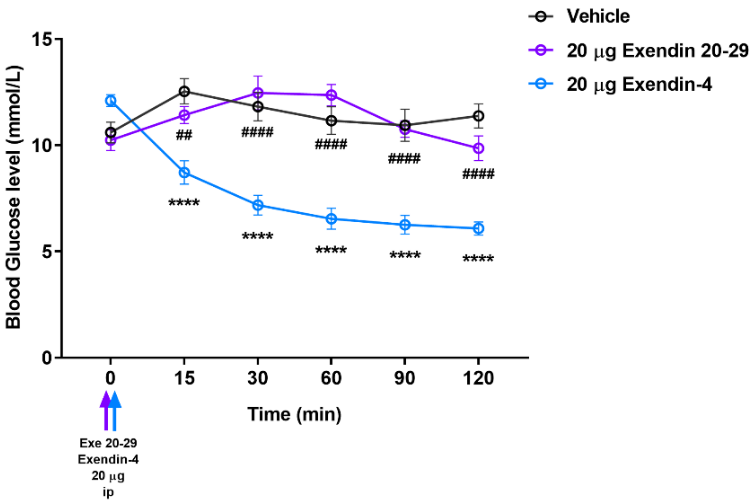


**Supplementary Fig. 8 Exendin 20–29 showed no effect on blood glucose levels with the mass effective for pain relief in mice.** Effects of intraperitoneal administration of 20 μg exendin 20–29 or 20 μg exendin-4 on blood glucose levels (mean ± S.E.M., *n* = 5). Two-way ANOVA followed by Bonferroni multiple comparison test (*****p* <.0001, compared with the vehicle group; ##*p* < 0.01, ####*p* <.0001, compared with 20 μg exendin-4 treatment). ANOVA: analysis of variance, S.E.M: standard error of mean.

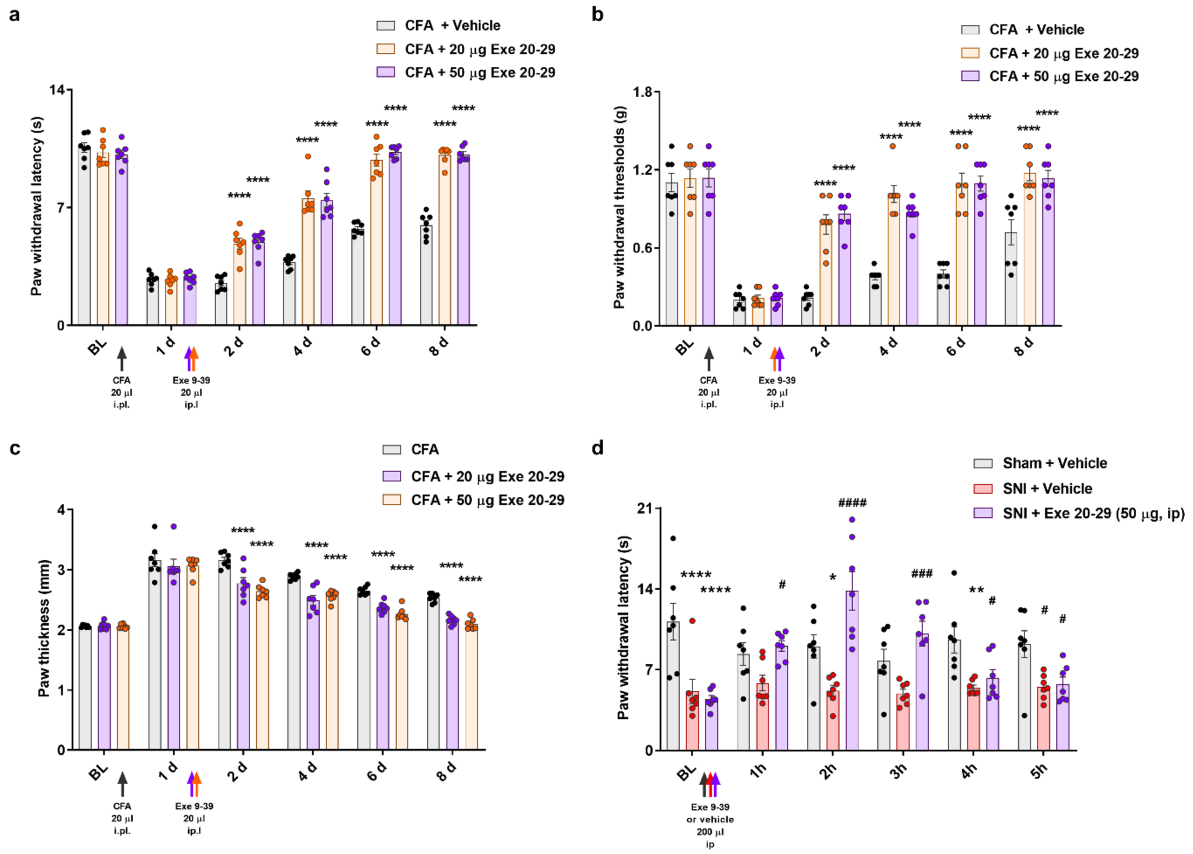


**Supplementary Fig. 9 Alleviation of CFA-induced inflammatory and SNI-induced neuropathic pain via exendin 20–29 administration. a** Effects of intraplantar injection of exendin 20–29 (Exe 9–39) (20 and 50 μg) on thermal hyperalgesia in CFA-induced inflammatory chronic pain mouse model using Hargreaves test (mean ± S.E.M., *n* = 7 each). Two-way ANOVA followed by Bonferroni multiple comparison test (*****p* <.0001, compared with the CFA group). **b** Effects of intraplantar injection of exendin 20–29 (Exe 20–29) (20 and 50 μg) on mechanical allodynia in CFA-induced inflammatory chronic pain mouse model using von Frey test (mean ± S.E.M., *n* = 7 each). Two-way ANOVA followed by Bonferroni multiple comparison test (*****p* <.0001, compared with the CFA group). **c** Reduced paw swelling and thickness via exendin 20–29 intraplantar administration in CFA-induced inflammatory pain mouse model. Measurement of the hind paw thickness (mm) on days 1, 2, 4, 6, and 8 following intraplantar administration of exendin 20–29 (Exe 20–29) (20 and 50 μg) (mean ± S.E.M., *n* = 7). Two-way ANOVA followed by Bonferroni multiple comparison test (****p <.0001, compared with the CFA group). **d** Effects of intraperitoneal injection of exendin 20–29 (Exe 9–39) (50 μg) on thermal

hyperalgesia in SNI-induced neuropathic chronic pain mouse model using Hargreaves test (mean ± S.E.M., $n = 7$ each). Two-way ANOVA followed by Bonferroni multiple comparison test (*$p < 0.05$, **$p < 0.01$, ****$p <.0001$, compared with the Sham + Vehicle group; #$p < 0.05$, ###$p <.001$, ####$p <.0001$, compared with the SNI + Vehicle group). ANOVA: analysis of variance, CFA: complete Freund's adjuvant, S.E.M: standard error of mean, SNI: spared nerve injury.

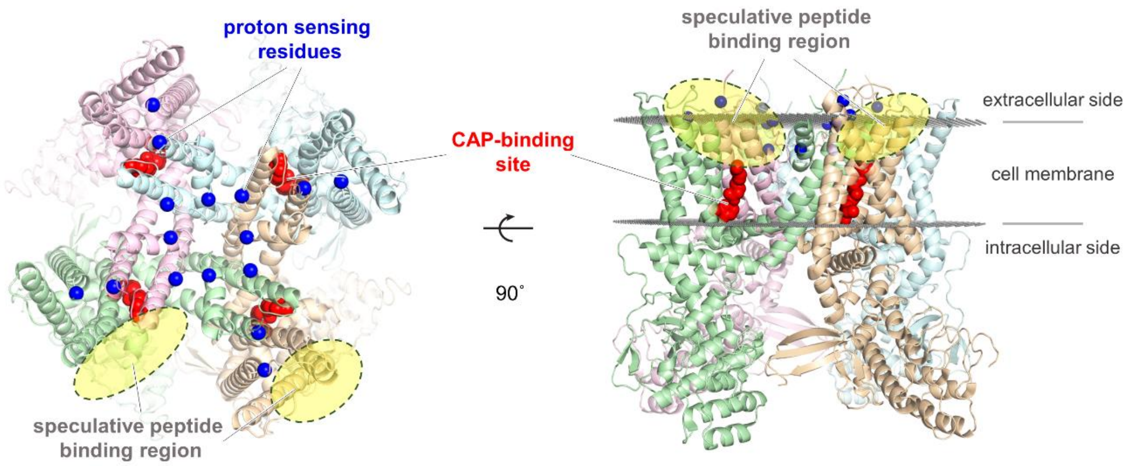


**Supplementary Fig. 10 Structural implication of interaction between TRPV1 and GLP-1 and its derived peptides.** Receptor binding implications showing the potential interactions regions of GLP-1 and its derived peptides with TRPV1 based on our results. The TRPV1 structure is based on the cryo-EM structure from human (PDB ID: 7L2H). Two views are provided: one from the extracellular side (left) and one rotated 90 degrees to show the cell membrane orientation (right). Proton sensing residues (human: E600, E648, R455, K639; mouse: E604, E652, R459, K643) are marked with blue spheres, and the CAP-binding site is marked in red. Yellow highlights denote speculative peptide binding areas. Note that the locations indicated are provisional and have not been validated through direct docking studies.
